# Identification, characterization, and gene expression analysis of nucleotide binding site (NB)-type resistance gene homologues in switchgrass

**DOI:** 10.1186/s12864-016-3201-5

**Published:** 2016-11-08

**Authors:** Taylor P. Frazier, Nathan A. Palmer, Fuliang Xie, Christian M. Tobias, Teresa J. Donze-Reiner, Aureliano Bombarely, Kevin L. Childs, Shengqiang Shu, Jerry W. Jenkins, Jeremy Schmutz, Baohong Zhang, Gautam Sarath, Bingyu Zhao

**Affiliations:** 1 Department of Horticulture, Virginia Tech, Blacksburg, VA 24061 USA; 2 Grain, Forage and Bioenergy Research Unit, USDA-ARS, Lincoln, NE 68583-0937 USA; 3 Department of Biology, East Carolina University, Greenville, NC 27858 USA; 4 Crop Improvement and Genetics Research, USDA-ARS, Albany, CA 94710 USA; 5 Department of Biology, West Chester University of Pennsylvania, Wester Chester, PA 19382 USA; 6 Department of Plant Biology, Michigan State University, East Lansing, MI 48824 USA; 7 Department of Energy Joint Genome Institute, Walnut Creek, CA 94595 USA; 8 HudsonAlpha Institute for Biotechnology, Huntsville, AL 35806 USA; 9 407 Latham Hall, 220 Ag Quad Lane, Blacksburg, VA 24061 USA

**Keywords:** Biofuel, Disease resistance, Gene expression, NB-LRR, *Panicum virgatum* (switchgrass), RNA-seq, SNP

## Abstract

**Background:**

Switchgrass (*Panicum virgatum* L.) is a warm-season perennial grass that can be used as a second generation bioenergy crop. However, foliar fungal pathogens, like switchgrass rust, have the potential to significantly reduce switchgrass biomass yield. Despite its importance as a prominent bioenergy crop, a genome-wide comprehensive analysis of NB-LRR disease resistance genes has yet to be performed in switchgrass.

**Results:**

In this study, we used a homology-based computational approach to identify 1011 potential NB-LRR resistance gene homologs (RGHs) in the switchgrass genome (v 1.1). In addition, we identified 40 RGHs that potentially contain unique domains including major sperm protein domain, jacalin-like binding domain, calmodulin-like binding, and thioredoxin. RNA-sequencing analysis of leaf tissue from ‘Alamo’, a rust-resistant switchgrass cultivar, and ‘Dacotah’, a rust-susceptible switchgrass cultivar, identified 2634 high quality variants in the RGHs between the two cultivars. RNA-sequencing data from field-grown cultivar ‘Summer’ plants indicated that the expression of some of these RGHs was developmentally regulated.

**Conclusions:**

Our results provide useful insight into the molecular structure, distribution, and expression patterns of members of the NB-LRR gene family in switchgrass. These results also provide a foundation for future work aimed at elucidating the molecular mechanisms underlying disease resistance in this important bioenergy crop.

**Electronic supplementary material:**

The online version of this article (doi:10.1186/s12864-016-3201-5) contains supplementary material, which is available to authorized users.

## Background

Switchgrass (*Panicum virgatum* L.) is a North American prairie grass that can be used as a second generation bioenergy feedstock. As a readily outcrossing species, native switchgrass germplasms have maintained a high level of genetic diversity over time [1, 2]. Two ecotypes of switchgrass, lowland and upland, have emerged that are adapted to different growth habitats. Lowland ecotypes are generally tetraploid in nature and grow in warm, moist, southern climates whereas upland ecotypes of switchgrass can be tetraploid, hexaploid, or octoploid and are usually found growing in the northern part of the United States into southern Canada [3]. Lowland ecotypes also typically produce more biomass and are more tolerant to diseases than their upland counterparts; however, upland ecotypes are considered to be more tolerant to drought and cold stresses [3, 4].

Currently, industrial scale breeding programs of switchgrass have focused on optimizing biomass yield and improving feedstock quality in order to produce more biofuel [5]. However, this practice is likely to reduce the genetic diversity in switchgrass that promotes disease resistance. Airborne foliar fungal pathogens, like switchgrass rust, have potential to cause nationwide epidemics on switchgrass and have been shown to cause significant biomass yield losses [6]. The causal agent of switchgrass rust, *Puccinia emaculata* Schw., is widespread and has been reported in Tennessee [7], Arkansas [8], Virginia [9], and Mississippi [10]. The majority of switchgrass cultivars, including lowland and upland ecotypes, have been shown to be moderate to highly susceptible to this rust pathogen [11, 12].

In order to understand and improve disease resistance in switchgrass, the molecular mechanisms underlying tolerance to pathogen infection must be elucidated. A recent report found that many genes were differentially expressed during switchgrass rust infection and that some of these genes belonged to the NB-LRR gene family, which is the largest family of plant disease resistance (*R*) genes [13]. NB-LRR genes encode proteins that contain a C-terminal leucine rich repeat (LRR) domain, a highly conserved central nucleotide binding (NB) domain, and a variable N-terminal region [14]. The NB domain has been well characterized with regards to several preserved motifs including the Walker-A/P-loop, kinase 2, RNBS/kinase 3, and GLPL [15]. Recent studies have suggested that the LRRs within the C terminal region may contain either a ‘LxxLxxLxxLxLxx’ signature [16] or a ‘LxxLxxL’ signature [17]. Two major classes of NB-LRR genes have emerged based on conserved sequences within the N-terminal. These classes are characterized by the presence of either a coiled-coil (CC) motif or a domain homologous to the *Drosophila* Toll and mammalian Interleukin-1 Receptor (TIR) [15]. To date, no TIR-NB-LRR genes have been identified in cereal grass species [18].

Putative NB-containing *R* genes have been identified in numerous plant species by experimental methods, such as PCR cloning [19] which has been used to identify potential RGHs in species such as Arabidopsis [20], rice [18], and cotton [21]. Homology-based bioinformatics approaches have also been used to identify thousands of putative NB-containing *R* genes in plants, including several important crop species such as rice [22], potato [23], and soybean [24]. A recent study by Li et al. [25] used readily available genomes for four grass species to computationally identify 129, 245, 239, and 508 NB-LRR *R* genes in maize, sorghum, Brachypodia, and rice, respectively. Alternatively, advances in next generation sequencing technologies have allowed for preferential high throughput sequencing of *R* genes in a process known as Resistance gene enrichment Sequencing (Ren-Seq) [26]. This method has been successfully used in potato and tomato to identify NB-LRR resistance genes [26].

Depending on the size of the plant genome, it is estimated that NB-LRR genes comprise on average between 0.5 and 1.8 % of the protein coding genes [15, 27]. A few plant species, such as cucumber [28] and papaya [29], have considerably less than 0.5 % NB-LRR genes. Additionally, NB-LRR genes in grass species have been calculated to make up anywhere from 0.4 to 1.35 % the total number of predicted proteins [25]. Despite recent advances in determining potential *R* genes, few studies have been performed in switchgrass. One recent report by Zhu et al. [30] identified RGHs in switchgrass using a combination of PCR-cloning and EST database mining. The results of this study identified approximately 380 RGHs. Since there are an estimated 98,007 protein-coding loci in the switchgrass genome [31], the number of RGHs identified by Zhu et al. [30] is roughly 0.39 % of the total number of protein-coding transcripts in switchgrass. The recent release of the draft switchgrass genome (v 1.1) provides an excellent opportunity to comprehensively identify NB-LRR RGHs in this important bioenergy plant. Using a homology-based computational method, 1011 putative NB-containing RGHs were identified in the switchgrass genome (v 1.1). We also identified several switchgrass RGHs that contained unique domains, including a jacalin-like lectin binding domain, a calmodulin-like binding domain, and a major sperm protein domain. Additionally, RNA-sequencing (RNA-seq) data from a rust-resistant cultivar, ‘Alamo’ [9], and a rust-susceptible cultivar, ‘Dacotah’ [9], was used to identify variants within the RGHs and to examine basal expression differences of these RGHs in uninfected leaf tissue. An analysis of RNA-seq datasets from the flag leaves of field-grown cultivar ‘Summer’ plants indicated that the expression levels of some RGHs were developmentally regulated. The results of this study significantly improve our understanding of disease resistance genes in this important biofuel crop species.

## Methods

### Switchgrass genome resources and identification of putative NB-LRR resistance genes

The switchgrass genome (*Panicum virgatum* v1.1, DOE-JGI) and its annotation resources were accessed from the DOE-JGI website (http://www.phytozome.net/panicumvirgatum.php) [31]. HMMER 3.0 (http://hmmer.org/) [32] and PfamScan (http://www.ebi.ac.uk/Tools/pfa/pfamscan/) were employed to search for switchgrass genes that contained Pfam NB (NB-ARC) family (PF00931) domains. The search was conducted using switchgrass protein sequences that are based on the curated Pfam-A dataset (Pfam10.0) [33] and the threshold was set to <1E-10. To predict LRRs, a Perl script was developed that identified and counted the number of ‘LxxLxxLxx’ signatures in the C-terminal region of the switchgrass RGHs.

### Structural analysis of the newly identified RGHs

Coils (v 2.2) [34], a program embedded in the InterProScan software (v 5.11) [35], was employed to search the putative switchgrass RGHs for the presence of CC domains. SMART (Simple Modular Architecture Research Tool, http://smart.embl.de/), a widely used online resource that identifies and annotates protein domains and protein domain architectures [36], was used to search the RGHs for TIR domains. The SMART and Pfam databases were also used to detect other highly conserved unique domains that may be present in the RGHs. SignalP (v 4.0) [37], as part of InterProScan, was used to analyze the N-terminal region of the putative switchgrass RGHs for the presence of signal peptides. TargetP 1.1 (http://www.cbs.dtu.dk/services/TargetP/) was then employed to analyze the subcellular localization of the switchgrass RGHs that contained a signal peptide [38]. Finally, NLStradamus [39] was used to determine if any of the RGHs may contain a putative nuclear localization signal.

### Phylogenetic analysis of switchgrass RGHs containing full-length NB domains

The amino acid sequences of the switchgrass RGHs were manually searched for the presence of 4 highly conserved motifs within the NB domain: the P-loop/WalkerA, the Kinase 2, the RNBS, and the GLPL [15]. For those RGHs in which all four motifs could be easily identified, the amino acid sequences were extracted and compiled together. Clustal Omega [40] was used to align the sequences and Skylign [41] was used to determine the consensus sequences for the four conserved motifs mentioned above. Sequences that closely followed the consensus sequence for each motif were kept for further analysis. To analyze the genetic diversity present in the switchgrass RGHs, the amino acid sequences of the full-length NB domains, starting from the beginning of the P-loop and ending at the GLPL motif, were compared to 116 full-length NB domains from *Brachypodium distachyon* [42]. All of the sequences were aligned using ClustalW, as part of the MEGA software (v 6) program, and the default parameters [43]. After alignment, a Maximum-likelihood phylogenetic tree was constructed in MEGA using all sites and the remaining default parameters and a bootstrap value of 100.

### Plant materials

The seeds for the ‘Alamo’ and ‘Dacotah’ plants used in this study were obtained from the USDA Plant Genetic Resources Conservation Unit (Griffin, Georgia). The plants were maintained in the greenhouse at Virginia Tech. The source of the field grown switchgrass plants were maintained at the experimental farms of the University of Nebraska, near Mead, NE [44].

### Plant growth conditions and tissue collection

One rust-resistant ‘Alamo’ plant and one rust-susceptible ‘Dacotah’ plant were used for this study. The plants were grown in the greenhouse facility at Virginia Tech with a 12–14 h photoperiod and day/night temperatures of 28 and 22 °C, respectively. After 3 months of growth, each plant was clonally propagated into four biological replicates by planting single tillers in Miracle-Gro Potting Mix (Miracle-Gro Lawn Products, Inc., Marysville, OH, USA) in 1.1 × 10 -2 m3 pots. The plants were watered twice a week and maintained in the greenhouse. After 2 months of growth, the first fully expanded leaf of E2-E3 stage tillers was collected for each biological replicate, frozen in liquid nitrogen, and stored at −80 °C until RNA isolation.

### RNA isolation and sequencing

Total RNA was isolated from switchgrass leaf samples using a modified TRIzol combined with columns method. Briefly, frozen leaf samples were ground to a fine powder in liquid nitrogen using a mortar and pestle. TRIzol reagent was added to the frozen tissue and total RNAs were extracted following the manufacturer’s protocol. After precipitation of the RNA with 100 % isopropanol, the mixture was applied to an RNeasy spin column that is part of the Qiagen RNeasy Plant Mini kit (Qiagen, Valencia, CA, USA). The remaining RNA isolation steps, including DNase treatment, were performed according to the manufacturer’s protocol.

After RNA isolation, the quality and quantity of the RNA was assessed using a Nanodrop-D1000 (Nanodrop, Wilmington, DE, USA) and a bioanalyzer (Virginia Bioinformatics Institute, Blacksburg, VA, USA). RNA for one sample of ‘Alamo’ and one sample of ‘Dacotah’ was sent to the Genomics Resources Core Facility at Weill Cornell Medical College (Cornell Medical School, New York, NY, USA) for 101 bp paired-end RNA-sequencing. The remaining six RNA samples (3 biological replicates per cultivar) were sent to the Genomics Facility of Michigan State University (Lansing, MI, USA) for 50 bp single-end RNA-sequencing.

### RNA-seq analysis and variant detection between ‘Alamo’ and ‘Dacotah’

The 101 bp paired-end RNA-seq datasets for ‘Alamo’ and ‘Dacotah’ were imported and analyzed using CLC Genomics Workbench (v 7.5). Raw reads that passed default quality scores and were greater than 50 bp in length were mapped to the switchgrass (v 1.1) reference genome (Table 2) using the RNA-seq analysis tool with the following parameters: mapping was also performed to intergenic regions, only 1 hit was allowed per read, a similarity fraction of 0.9, a length fraction of 1.0, a mismatch cost of 2, an insertion cost of 3, and a deletion cost of 3.

After read-mapping, the reads that mapped were locally realigned and variants were called using the Basic Variant Detection tool with the following parameters: broken pairs were not ignored, non-specific matches were ignored, minimum read coverage of 3, minimum variant count of 2, and a minimum variant frequency (%) of 25.0. Following variant detection, the variant tracks for both ‘Alamo’ and ‘Dacotah’ were filtered against the RNA-seq mapped reads of the other cultivar, respectively, to identify variants uniquely different between them. High-quality variants, including single nucleotide polymorphisms (SNPs), multiple nucleotide polymorphisms (MNPs), insertions/deletions, and replacements were selected for based on the following criteria: zygosity = homozygous, Frequency = 100 %, coverage ≥3, control count = 0, control coverage ≥3, and control frequency = 0 %. The resulting lists from both variant detections were compiled together for a comprehensive list of high quality variants between the two cultivars (Additional file 1: Table S1).

### Gene expression analysis of switchgrass RGHs in ‘Alamo’, ‘Dacotah’, and ‘Summer’

In order to determine if any of the RGHs identified in this study are expressed basally in switchgrass leaves, the 50 bp single-end RNA-Seq datasets for the three biological replicates of ‘Alamo’ and ‘Dacotah’ were analyzed using CLC Genomics Workbench. Reads with a quality score (Q-score) >20 were mapped to the switchgrass reference genome (v 1.1) (Table 2). After read-mapping, the gene expression output tracks for all replicates of ‘Alamo’ and ‘Dacotah’ were compared using readily available tools within the program. A gene was considered expressed if five or more total reads mapped to it. Differential expression analysis between the two cultivars was carried out using an Empirical analysis of Differential Gene Expression (EDGE) test that is available as part of the CLC Genomics Workbench software. The results were filtered based on a False Discovery Rate (FDR) *p*-value of < 0.05 and a corrected fold change >2.

We utilized the raw RNA-sequencing data obtained from flag leaves of field grown cultivar ‘Summer’ plants [44] to analyze developmental gene expression of the 1011 switchgrass RGHs over the course of a growing season. Only RGHs that exhibited detectable expression levels in all three biological replicates, and also in at least one of the five sampled time points, were kept for further analysis. Weighted Gene Co-expression Network Analysis (WGCNA, version 1.43) in R was then used to identify RGHs that demonstrated similar expression patterns [45–47]. Signed co-expression networks were identified using WGCNA with a soft threshold value of 16 and a minimum module size of 30. The module eigengene (ME), which corresponds to the first principal component of a specific module, represents the expression pattern of each co-expression module.

### Isolation of DNA from ‘Alamo’ and ‘Dacotah’

DNA was isolated from young leaf tissue using a modified CTAB method [48]. The DNA was re-suspended in 1x TE buffer (10 mM Tris pH 8.0, 10 mM EDTA pH 8.0) and the quantity and quality was measured using a Nanodrop ND-1000 (Wilmington, DE, USA).

### Validation of SNPs using allele-specific PCR primers and DNA sequencing

Allele-specific SNP PCR primers for both ‘Alamo’ and ‘Dacotah’ were designed as previously described [49]. Both the forward and reverse primers for each locus accounted for a SNP at the 3′ end. The 3^rd^ base pair from the 3′ end of each primer was changed according to the recommendations from Hirotsu et al. [49] in order to maximize allele-specificity. Four sets of forward and reverse primers that detected 16 SNPs were designed and used for PCR amplification in a total reaction volume of 20 μL (Additional file 2: Table S2). After the reactions were completed, the products were separated by gel electrophoresis on a 1.0 % agarose gel and visualized using a GelDoc system (Bio-Rad, Hercules, CA, USA).

Conserved PCR primers were also designed to amplify DNA fragments containing SNPs between ‘Alamo’ and ‘Dacotah’ (Additional file 3: Table S3). These SNPs were validated using traditional DNA sequencing. PCR reactions were performed in a total volume of 30 μL and contained the following components: 15 μL of high fidelity iProof (Bio-Rad, Hercules, CA, USA), 11 uL of ddH_2_O, 2 μL of 200 ng/μL DNA, 1 μL of 10 μM forward primer, and 1 μL of 10 μM reverse primer. The conditions for the PCR reactions were: 98 °C for 3 min, followed by 30 cycles of denaturation at 98 °C for 30 s, annealing at 60 °C for 45 s, extension at 72 °C for 1 min and 30 s, and a final extension at 72 °C for 7 min. Once the PCR reactions finished, 2 μL of each reaction was run on a 0.8 % agarose gel in order to verify amplification. Next, the remaining 28 μL of each reaction was purified. Finally, all purified PCR products were sent for DNA sequencing.

### Availability of data and materials

The data and datasets supporting the conclusions of this article are included within the article and its supplemental files. In addition, the RNA-seq datasets used in this article have been deposited in the GenBank database and can be found under the following accession numbers: SRR3473343, SRR3473344, SRR3467193, SRR3467194, SRR3467195, SRR3467196, SRR3467197 and SRR3467198. The phylogenetic tree generated in this study has been deposited in TreeBASE (Submission 19789) and can be accessed at the following URL: http://purl.org/phylo/treebase/phylows/study/TB2:S19789.

## Results

### Identification of 1011 putative NB-containing RGHs in switchgrass

**Fig. 1:**
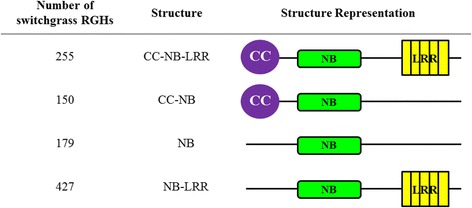
Numerical and structural representation of the 1011 switchgrass RGHs identified in this study. Four different protein structures were found for the 1011 switchgrass RGHs identified in this study. The coiled-coil (CC) domain is depicted in purple, the nucleotide binding (NB) domain in *green*, and the leucine rich repeat (LRR) region in *yellow*. The placement of the domains below does not reflect accurate molecular distances

In this study, a total of 1542 switchgrass proteins were identified that contained *R* gene-like NB-ARC domains. After selecting for protein sequences with an NB-ARC e-value of <1E-10 and removing alternatively spliced transcripts, a final number of 1011 unique switchgrass proteins were collected that contained *R* gene-like NB-ARC domains (Fig. 1, Additional file 1: Table S1). This accounts for approximately 1.03 % of the total protein-coding genes in switchgrass. From here on, these will be referred to as putative switchgrass RGHs. The putative RGH proteins varied in length from 98 amino acids (Pavir.J31808) to 1649 amino acids (Pavir.Ba02898). Of the 1011 RGH proteins, 695 are considered to be complete protein sequences in the switchgrass genome (v 1.1), meaning they are predicted to contain a start methionine (ATG) and a stop codon. The remaining 316 RGH proteins lacked one or both of these features. All of the 1011 putative switchgrass RGHs were manually annotated to validate the presence of the NB-ARC domain (Additional file 4: File S1).

**Fig. 2:**
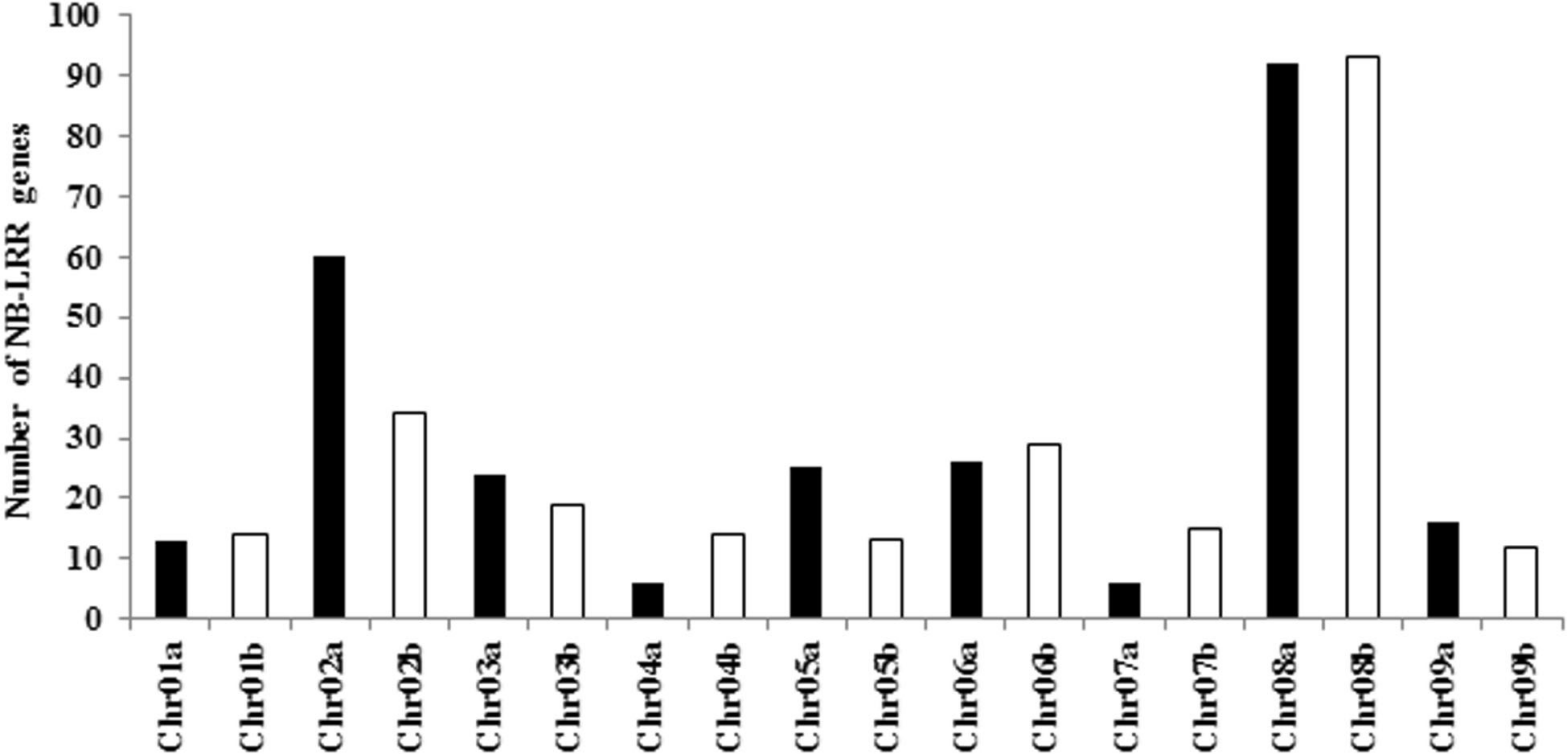
Chromosomal distribution of the 1011 switchgrass RGHs identified in this study. The switchgrass A and B sub-genomes are marked as *black and white*, respectively. An additional 500 genes were found on unanchored contigs (data not shown)

Since NB-LRR disease resistance genes tend to cluster together in plant genomes [15], the availability of a draft reference genome for switchgrass provided an opportunity to analyze the chromosomal distribution of the 1011 RGHs. The current version of the switchgrass genome (v 1.1) is comprised of 18 main pseudomolecules that represent the A and B subgenomes [31]. An additional several hundred thousand sequences are located on unanchored contigs [31]. Of the 1011 RGHs identified in this study, 511 were assigned to one of the major pseudomolecules while the remaining 500 were dispersed among unanchored contigs (Fig. 2). Switchgrass chromosome 8 was found to contain the most RGHs with 92 genes located on Chr08a and 93 genes located on Chr08b. In contrast, chromosomes 4 and 7 contained the least total number of RGHs with 20 and 21, respectively (Fig. 2).

### Structural analysis of the 1011 newly identified RGHs in switchgrass

The two major classes of NB-LRR disease resistance genes contain either a coiled-coil (CC) motif or a domain homologous to the intracellular signaling domain of *Drosophila* Toll and mammalian Interleukin-1 Receptors (TIR) in their N-terminals [14]. A total of 405 genes were discovered that contain putative CC domains (Fig. 1, Additional file 2: Table S2). In contrast to the CC domains, no TIR domains were detected in any of the 1011 RGHs.

Using SignalP, 19 of the 1011 switchgrass RGHs were identified to contain an N-terminal signal peptide (Additional file 3: Table S3). None of these signal peptides were predicted to span a cellular membrane. Based on the amino acid sequence of the signal peptide, TargetP (v 1.1) was used to predict the subcellular localization of these proteins (Additional file 3: Table S3). Of the 19 proteins, 13 were predicted to enter the secretory pathway and 4 were predicted to go to the mitochondria. The remaining proteins, Pavir.Ea02039.1 and Pavir.Ba00602.1, were predicted to go to other subcellular locations that were not the mitochondria or the chloroplast. Several NB-LRR disease resistance proteins, such as RRS1 [50], have been shown to localize in the plant nucleus upon pathogen detection. NLStradamus was employed to search the 1011 RGHs for the presence of putative nuclear localization signals (NLSs). A total of 104 of the 1011 RGHs were identified to contain a putative NLS (Additional file 5: Table S4). Of these proteins, the NLS was predicted to be in the N-terminal region in 72 of the RGHs, while 32 of the proteins were predicted to contain an NLS in their C-terminal region.

The majority of NB-containing resistance genes contain highly variable leucine rich repeats (LRRs) in their C-terminals. LRRs are believed to function in protein-protein interactions, as well as in ligand binding [14]. The 1011 switchgrass RGH proteins were manually screened to identify a consensus sequence (LxxLxxLxx) that was predominant among the RGH proteins. A Perl script determined that a total of 682 of the 1011 switchgrass RGHs contained one or more ‘LxxLxxLxx’ signatures downstream of the end of the NB-ARC domain (Fig. 1, Additional file 6: Table S5). The number of ‘LxxLxxLxx’ signatures identified ranged from 1 to 19 per protein with the majority of these proteins containing between 5 and 9 (445 or 65.2 %). Therefore, the majority of the switchgrass RGHs do in fact have the typical NB-LRR gene structure.

### Identification of unique domains in the 1011 switchgrass RGHs

Previous reports have identified unique domains in the N- and C-terminals of NB-LRR resistance genes and have suggested that these domains may play a role in *R* gene function [51]. As summarized in Table 1, 40 switchgrass NB-LRR proteins were also predicted to contain other known functional domains. These unique domains can be classified into 7 different functional categories: protein modification, DNA binding/transcription, protein trafficking and vesicle movement, protein-protein interaction, sugar binding, signal transduction, and transposable element. The majority of switchgrass RGHs with unique domains (24 out of 40, or 60 %) fell into the protein modification category. Of these 24 proteins, two RGHs contained a thioredoxin domain (Pavir.Fa01782 and Pavir.Hb00484), one RGH contained a glutaredoxin domain (Pavir.Bb01048), and one RGH contained a phosphatase domain (Pavir.J24356). A putative C-terminal NLS was also detected for Pavir.Fa01782. Interestingly, the remaining 20 RGHs were found to contain a protein kinase domain (Table 1). Of these 20 proteins, ten are located on chromosome 8 (5 on Chr08a and the other 5 on Chr08b). Two of these protein kinase-containing RGHs, Pavir.Ha00561 and Pavir.Ha01108, were also found to contain a NLS in their C-terminal and N-terminal, respectively.

**Table 1:** Unique domains that were identified in 40 switchgrass NB or NB-LRR proteins

Category	Unique domain	Protein ID	E-value	Structure
Protein modification	Glutaredoxin	Pavir.Bb01048.1.p	9.80E-08	NB-LRR-Glutaredoxin
	Thioredoxin	Pavir.Fa01782.1.p	3.70E-22	NB-LRR-Thioredoxin
		Pavir.Hb00484.1.p	8.50E-21	NB-LRR-Thioredoxin
	Protein tyrosine kinase	Pavir.Ba02898.1.p	1.50E-26	NB-LRR-PK
	Serine/threonine phosphatases, family 2C, catalytic domain	Pavir.J24356.1.p	2.90E-14	NB-LRR-PP2C
	Serine/Threonine protein kinases, catalytic domain	Pavir.Aa03320.1.p	2.30E-13	PK-NB-LRR
		Pavir.Bb00833.1.p	3.20E-30	PK-NB
		Pavir.Ha00561.1.p	4.10E-26	PK-NB-LRR
		Pavir.Ha00691.1.p	2.10E-97	NB-LRR-PK
		Pavir.Ha01101.1.p	2.40E-23	PK-NB-LRR
		Pavir.Ha01108.1.p	1.70E-25	PK-NB-LRR
		Pavir.Ha01246.1.p	4.00E-21	PK-NB-LRR
		Pavir.Hb00345.1.p	7.80E-16	PK-NB-LRR
		Pavir.Hb00951.1.p	6.20E-28	PK-NB
		Pavir.Hb01026.1.p	3.10E-22	PK-NB
		Pavir.Hb01049.1.p	8.40E-13	PK-NB
		Pavir.Hb01188.1.p	1.40E-28	PK-NB
		Pavir.J00375.1.p	2.40E-25	PK-NB-LRR
		Pavir.J01180.1.p	4.60E-27	PK-NB-LRR
		Pavir.J16540.1.p	1.60E-23	PK-NB-LRR
		Pavir.J23132.1.p	7.30E-19	PK-NB
		Pavir.J36755.1.p	3.30E-12	PK-NB
		Pavir.J37596.1.p	1.30E-14	PK-NB
		Pavir.J39176.1.p	4.00E-14	PK-NB
DNA binding, transcription	B3 DNA binding domain	Pavir.Ba02315.1.p	2.20E-10	B3-NB-LRR
	B3 DNA binding domain	Pavir.J20878.1.p	3.10E-07	B3-NB-LRR-WRKY
	WRKY DNA binding domain		1.00E-14	
	BED zinc finger	Pavir.Fa02339.1.p	5.40E-09	ZF-NB-LRR
		Pavir.Ga00028.1.p	5.40E-09	ZF-NB-LRR
		Pavir.Gb00931.1.p	1.70E-08	ZF-NB-LRR
		Pavir.J19380.1.p	2.50E-12	ZF-NB-LRR
		Pavir.J40131.1.p	7.80E-08	ZF-NB
	WRKY DNA-binding domain	Pavir.J33941.1.p	1.80E-14	NB-LRR-WRKY
protein trafficking and vesicle movement	MSP (Major sperm protein) domain	Pavir.Ib02384.1.p	1.10E-09	MSP-NB-LRR
		Pavir.Ib02433.1.p	1.50E-08	MSP-NB-LRR
		Pavir.J18369.1.p	5.10E-13	MSP-NB
Protein-protein interaction	WD domain, G-beta repeat	Pavir.J03445.1.p	5.00E-06	NB-LRR-WD
	hAT family C-terminal dimerisation region	Pavir.Fb01504.1.p	3.50E-08	NB-LRR-hAT
Sugar binding	Jacalin-like lectin domain	Pavir.Hb01174.1.p	5.60E-11	NB-LRR-Jacalin
Signal transduction	Calmodulin binding protein-like	Pavir.Hb00190.1.p	5.10E-79	NB-LRR-Calmodulin
Transposon element	gag-polypeptide of LTR copia-type	Pavir.Aa01444.1.p	8.90E-07	NB-LRR-LTR

The next largest category included RGHs with domains that function in DNA binding and transcription. Two switchgrass RGHs, Pavir.Ba02315 and Pavir.J20878, were found to contain an N-terminal B3 DNA binding domain. Interestingly, Pavir.J20878 is also predicted to contain a C-terminal WRKY domain. Five other RGHs including Pavir.Fa02339, Pavir.Ga00028, Pavir.Gb00931, Pavir.J19380, and Pavir.J40131 were also found to have an N-terminal zinc finger-BED DNA binding domain (Table 1).

Finally, the remaining eight switchgrass RGHs fell into the last 5 categories. Three switchgrass RGHs (Pavir.Ib02384, Pavir.Ib02433, and Pavir.J18369) were predicted to contain an N-terminal domain homologous to major sperm protein (PF00635) (Table 1). These proteins are classified in the protein trafficking and vesicle movement category. Two switchgrass RGHs were predicted to contain domains that may play a role in protein-protein interactions (Table 1). These include Pavir.J03445, which has a C-terminal WD40 domain, and Pavir.Fb01504, which contained a C-terminal hAT family dimerization region that is common to transposable elements. Pavir.J03445 was also predicted to contain an N-terminal nuclear localization signal. Another element that is found in some transposons, a gag-polypeptide of LTR copia-type domain, was predicted in the C-terminal of Pavir.Aa01444. The final two RGHs with unique domains, Pavir.Hb01174, which contains a C-terminal jacalin-like lectin binding domain, and Pavir.Hb00190, which has calmodulin binding protein-like domain in the C-terminal, are predicted to function in sugar binding and signal transduction, respectively.

### Phylogenetic analysis of switchgrass sequences containing a full-length NB domain

**Fig. 3:**
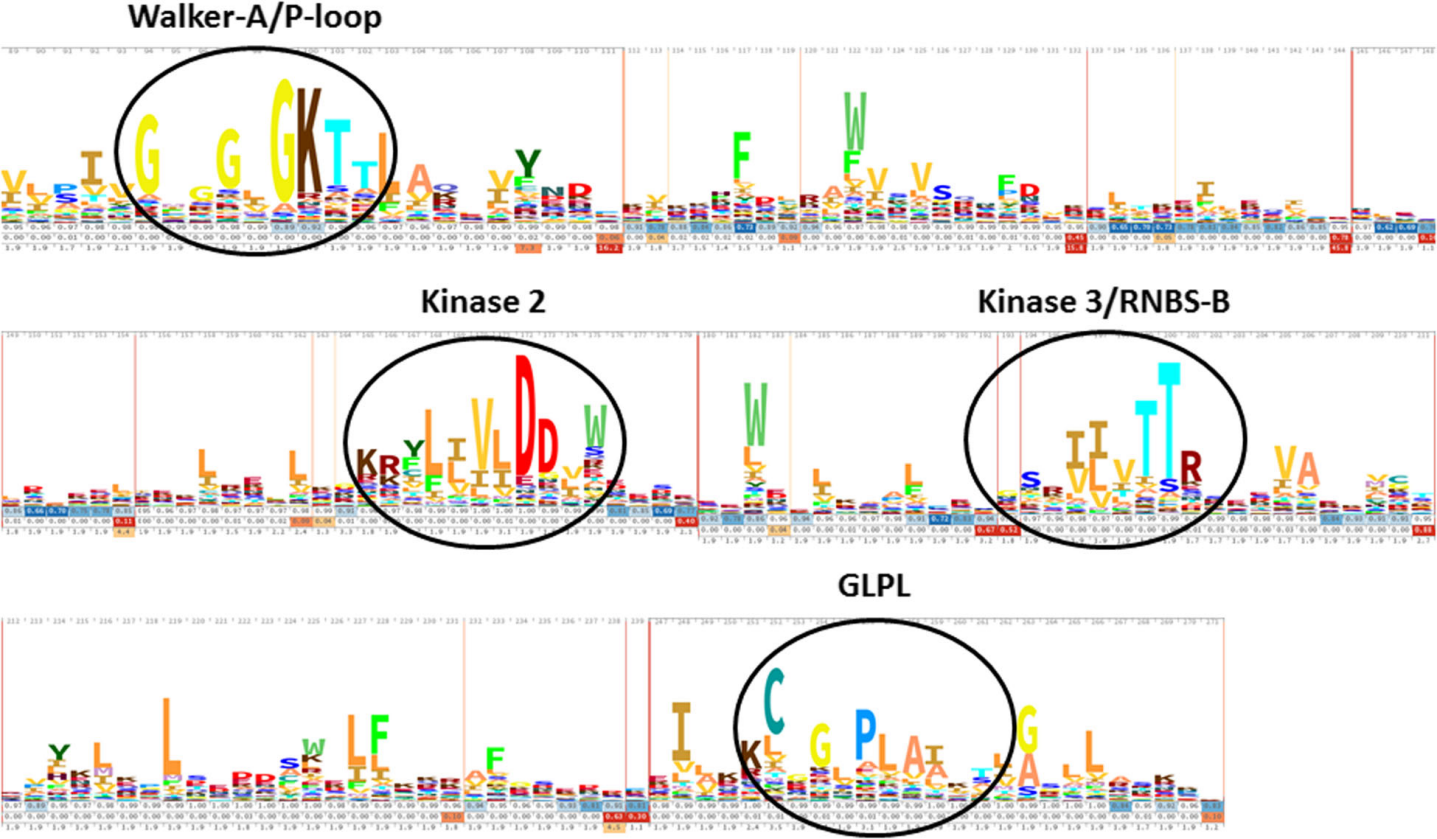
Skylign output of the NB region for the 600 switchgrass sequences that contained a full-length NB domain. Four highly conserved motifs were identified in these sequences: the Walker-A/P-loop motif, the Kinase 2 motif, the Kinase 3/RNBS-B motif, and the GLPL motif. The height of each letter within a stack corresponds to the frequency and conservation of that letter at that position

Of the 1011 switchgrass RGHs, 600 were found to contain a full-length NB domain in which four highly conserved motifs could be found: the Walker-A/P-loop, the Kinase 2, the Kinase 3/RNBS-B, and the GLPL. These 600 sequences were then aligned to determine the consensus sequences for these four motifs (Fig. 3). The consensus sequences for the four motifs are as follows: 1) Walker-A/P-loop = GxGGxGKT, 2) Kinase 2 = KR(Y/F)L(I/L)VLDD(V/L)W, 3) Kinase 3/RNBS-B = SR(I/V) (I/L)VTTR, and 4) GLPL = GLPLA. These results are consistent with similar sequences identified in NB-LRR genes in other plant species, such as rice [52] and *Brachypodium* [42].

**Fig. 4:**
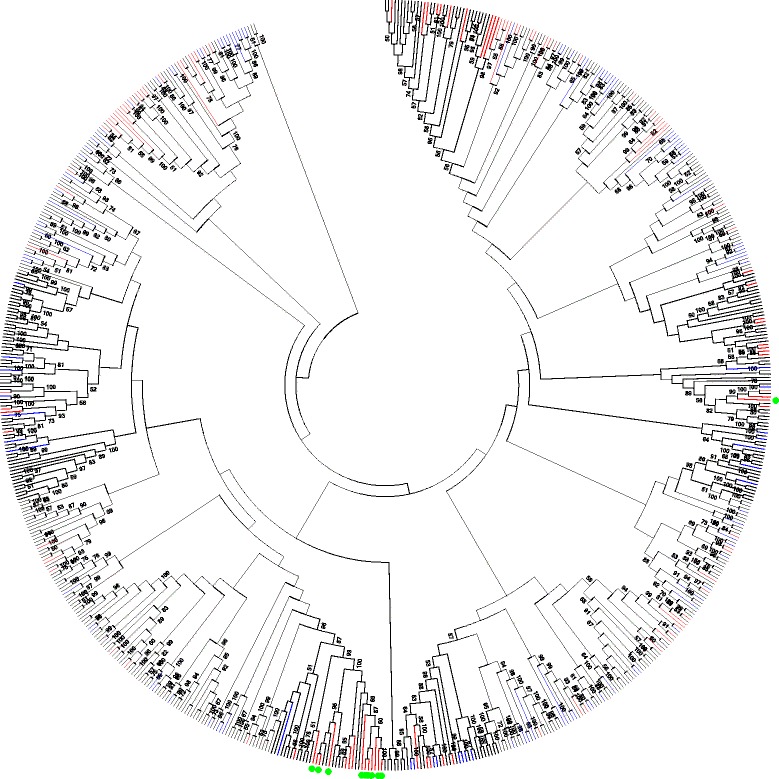
Phylogenetic analysis of 578 switchgrass RGHs and 116 *Brachypodium distachyon* RGHs. The phylogenetic tree was built using MEGA 6 with default settings. Branches for the *Brachypodium* RGHs are highlighted in *blue* and branches for switchgrass RGHs located on chromosome 8 are highlighted in *red*. The *green dots* represent the phylogenetic location for the 10 protein kinase-containing NB-LRR genes that are found on chromosome 8. Numbers on the tree indicate branches with bootstrap values greater than 50

After generating the consensus sequences for the four highly conserved motifs, only 578 switchgrass RGHs were found to closely match these sequences. The amino acid sequences for these RGHs, along with those of 116 *Brachypodium* NB-LRR genes [42], were extracted and then subjected to phylogenetic tree analysis. The un-rooted phylogenetic tree can be divided into 35 different groups, with the majority of groups containing both switchgrass and *Brachypodium* NB-LRR genes (Fig. 4, Additional file 7: Figure S1). Switchgrass and *Brachypodium* diverged about 50 million years ago [53]. However, most NB-LRR genes are still conserved in the two plant species, since no clear separation of switchgrass or *Brachypodium* genes clusters were observed (Fig. 4, Additional file 7: Figure S1). This is also supported by strong bootstrap values (>50) of many clades that included sequences from both species.

Switchgrass NB-LRR genes are enriched on chromosome 8 (Fig. 2). Therefore, we examined if chromosome 8 NB-LRR genes (highlighted in red) tended to cluster together in the phylogenetic tree (Fig. 4). As shown in Fig. 4, four small clusters of chromosome 8 NB-LRR genes are evident. These clusters were further analyzed to determine if the ten chromosome 8 RGHs that were predicted to contain a protein kinase domain were phylogenetically similar. Seven of the 10 protein kinase-containing RGHs (highlighted in green) were located in the same cluster, indicating that these genes are highly homologous. These genes could have rapidly evolved from the same ancestor and duplicated on chromosome 8. Interestingly, no *Brachypodium* genes were located in this cluster. The remaining three protein kinase-containing RGHs were dispersed across the phylogenetic tree with two of the genes clustering together in the same group as the bigger cluster. A similar trend of genes on the same chromosome clustering together was also observed for the *Brachypodium* genes (Fig. 4, Additional file 7: Figure S1).

### Identification of variants between RGHs in ‘Alamo’, a rust-resistant switchgrass cultivar, and ‘Dacotah’, a rust-susceptible switchgrass cultivar

The RNA-seq mapping statistics obtained from the analysis of these datasets is shown in Table 2. Approximately 99,283,683 ‘Dacotah’ reads (82.03 %) and 180,996,166 ‘Alamo’ reads (84.35 %) were mapped to the reference genome (Table 2). The slightly higher number of ‘Alamo’ reads mapping to the reference genome could be attributed to the fact that the switchgrass reference genome is a plant from the cultivar ‘Alamo’ (AP13).

**Table 2:** RNA-seq analysis of ‘Alamo’ and ‘Dacotah’ used for variant detection and gene expression analysis. One sample of ‘Alamo’ and one sample of ‘Dacotah’ were sequenced at a deep coverage (36X and 20X, respectively) and were used for variant detection. Three biological replicates of ‘Alamo’ and ‘Dacotah’ were used for gene expression analysis and were sequenced between 3.7X and 5.2X coverage

Sample	Sequencing	Initial reads	Trimmed reads	Mapped reads	% Reads mapped
Alamo	101 bp-PE	221,647,550	216,244,584	180,996,166	84.35
Dacotah	101 bp-PE	125,257,298	121,958,884	99,283,683	82.03
Alamo- 1	50 bp-PE	56,036,894	54,211,026	52,494,171	96
Alamo- 2	50 bp-PE	45,931,626	44,044,084	42,657,586	96
Alamo- 3	50 bp-PE	58,312,466	55,472,698	53,590,453	96
Dacotah- 1	50 bp-PE	59,434,186	56,774,376	54,489,840	96
Dacotah- 2	50 bp-PE	48,408,374	46,266,102	44,525,787	96
Dacotah- 3	50 bp-PE	63,304,978	61,060,688	58,815,617	96

After alignment to the switchgrass reference genome (v 1.1), the RNA-seq reads for both ‘Alamo’ and ‘Dacotah’ were analyzed in order to identify variants, including SNPs, MNPs, insertions/deletions (indels), and replacements in the RGHs. A total of 23,156 variants were found in 781 RGHs between ‘Alamo’ and ‘Dacotah’. After filtering for high quality homozygous variants, 2634 variants were found in 344 RGHs. These variants include 2347 SNPs, 136 MNPs, 145 indels, and 6 replacements (Additional file 8: Table S6).

**Fig. 5:**
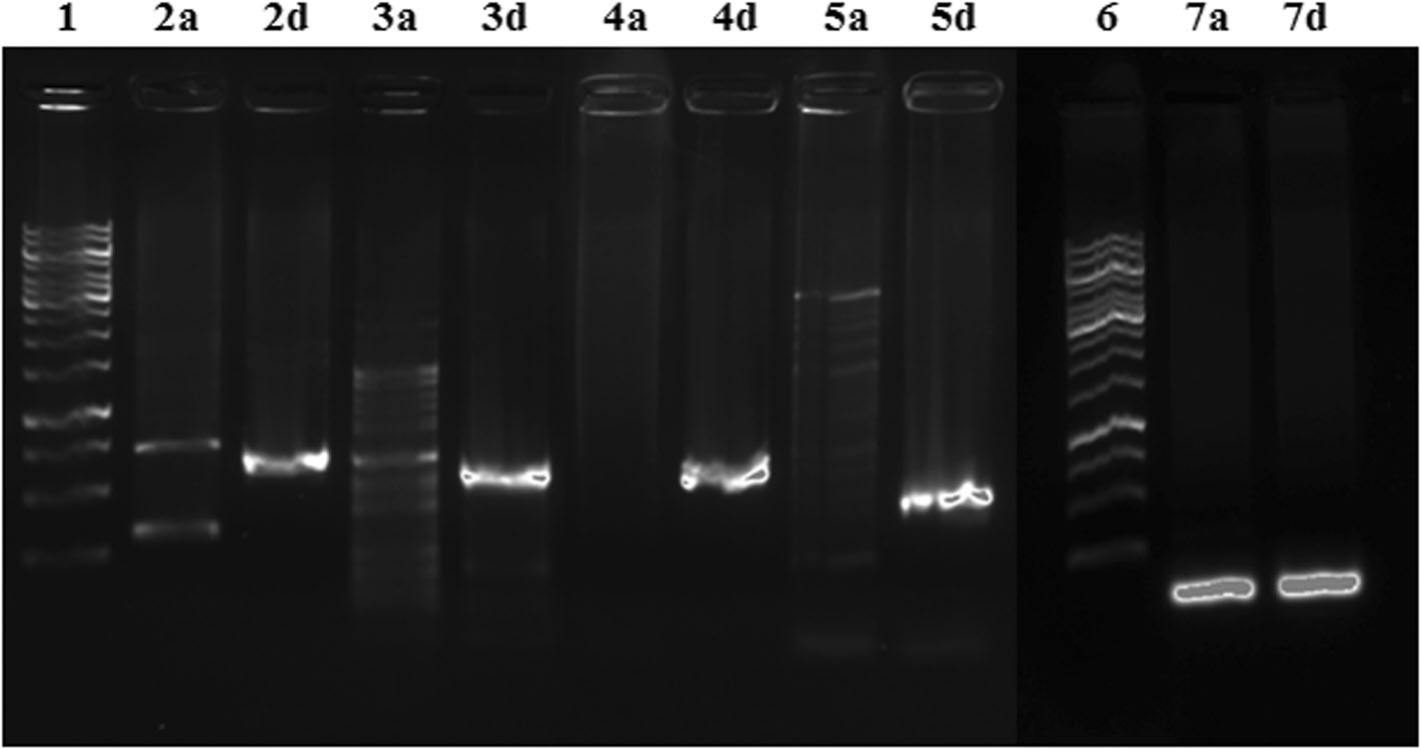
Validation of SNPs identified by RNA-sequencing using allele-specific primers. Pictured are the PCR products using ‘Dacotah’-specific primers where a = ‘Alamo’ DNA and d = ‘Dacotah’ DNA. 1) 1 kb ladder, 2) Pavir.Ba03659, 3) Pavir.Ba02315, 4) Pavir.Hb01688, 5) Pavir.Hb00487, 6) 1 kb ladder, 7) ELF1α

Allele-specific PCR is a high throughput and cost-effective way of distinguishing SNPs at a particular locus without the need for DNA sequencing [49]. A total of 8 primer pairs, 4 for ‘Alamo’ and 4 for ‘Dacotah’ (Additional file 9: Table S7), were designed to validate 16 SNPs identified by RNA-seq analysis. The results showed that the primers designed to detect the ‘Dacotah’ alleles were more specific to ‘Dacotah’ DNA than the primers designed to detect the ‘Alamo’ alleles (Fig. 5). The presence of unwanted PCR products using these allele-specific primers suggests that the primers may be amplifying unwanted PCR products. Alternatively, the other allele may actually be present in each DNA sample but is either not expressed or expressed at a level not captured by the sequencing depth of this experiment and thus, was not detected in our RNA-sequencing data.

Traditional PCR amplification and DNA sequencing with conserved primers was also used to validate SNPs identified from RNA-seq analysis. A total of 8 different primer sets were designed to detect 12 SNPs within 8 putative RGHs (Additional file 10: Table S8). Using this method, DNA sequencing confirmed the presence of 11 out of the 12 SNPs (Additional file 11: Figure S2). For the one SNP in Pavir.Ib01513 that could not be validated, the PCR products for the ‘Alamo’ and ‘Dacotah’ sequences differed greatly from the expected DNA sequence, indicating that this primer set may have amplified unwanted targets.

### Analysis of gene expression of the 1011 switchgrass RGHs between ‘Alamo’ and ‘Dacotah’

It has been suggested that NB-LRR disease resistance genes are basally expressed in plant tissues [54]. Upon pathogen detection, the expression of these genes is up-regulated in order to initiate defense responses [55]. RNA-sequencing reads from three biological replicates of ‘Alamo’ and ‘Dacotah’ (Table 2) were used to determine if any of the 1011 switchgrass RGHs were basally expressed in un-inoculated leaf tissue. Of the 1011 RGHs, 338 were expressed in both cultivars (Additional file 12: Table S9). For the individual cultivars, 117 RGHs were found to be expressed only in ‘Alamo’ and 134 RGHs were found to be expressed only in ‘Dacotah’ (Additional file 13: Table S10).

Differential expression of NB-LRR resistance genes has been shown to play a role in plant disease response. The results of gene expression analysis were filtered such that the 338 genes expressed in both cultivars were considered significantly differentially expressed if their RPKM values had a fold change greater than 2 and an FDR p-value less than 0.05. Using these criteria, 21 genes were found to be significantly differentially expressed between the two cultivars (Table 3). Overall, the results of this analysis suggest that these two different cultivars basally express RGHs.

**Table 3:** Significant Differentially Expressed RGHs between ‘Alamo’ and ‘Dacotah’

Gene ID	Chromosome	Gene start location	Gene end location	Alamo RPKM^a^means	Dacotah RPKM^a^means	Fold change^b^	FDR corrected *p*-value^3^
Pavir.Ha01577	Chr08a	45819137	45820543	0.074	11.565	54.837	6.03E-08
Pavir.Hb00191	Chr08b	3683280	3690800	22.965	4.363	−5.724	1.02E-07
Pavir.J41099	contig99714	31	3755	10.721	0.387	−23.222	6.52E-07
Pavir.Ha00254	Chr08a	5275668	5283177	8.387	0.088	−43.065	2.74E-06
Pavir.Ea02999	Chr05a	52240105	52245554	7.763	0.145	−31.66	9.51E-06
Pavir.J18698	contig20439	2509	7946	0.019	10.273	68.028	1.57E-05
Pavir.J36183	contig58243	960	5090	7.474	0.014	−57.923	1.77E-05
Pavir.Ha00258	Chr08a	5331659	5334691	12.753	0.617	−18.44	3.17E-05
Pavir.Ba03626	Chr02a	73291071	73293182	0.139	8.63	30.634	5.28E-05
Pavir.J38310	contig74920	865	4419	0.377	8.784	16.208	5.28E-05
Pavir.J13560	contig156657	131	2675	0.565	8.893	11.899	1.71E-04
Pavir.J35611	contig54273	2061	5449	0.195	5.96	17.525	4.86E-03
Pavir.J01215	contig01325	2932	5562	7.052	1.095	−6.492	5.22E-03
Pavir.J03054	contig03762	12948	18535	4.087	0.096	−20.509	7.55E-03
Pavir.Aa01110	Chr01a	14217597	14230708	3.764	0.03	−26.75	0.013
Pavir.Ha01146	Chr08a	31223571	31227884	0.703	5.777	6.478	0.019
Pavir.Cb00441	Chr03b	7318929	7322714	11.648	26.964	2.068	0.034
Pavir.Bb00035	Chr02b	557471	559270	0.054	3.333	18.041	0.041
Pavir.Ha00828	Chr08a	23240515	23246499	3.354	0.02	−25.367	0.041
Pavir.J29227	contig33532	301	4214	3.316	0.035	−22.844	0.041
Pavir.J02637	contig03134	4596	14505	4.903	0.772	−6.169	0.048

^a^ *RPKM* reads per kilobase per million of reads mapped

^b^Fold Change = expression level difference between the two samples when using ‘Dacotah’ as the reference sample

^3^FDR corrected *p*-value = *p*-value that the Fold Change is significant based on the corrected False Discovery Rate

### Expression of specific switchgrass NB-LRR genes are regulated by leaf developmental stages

**Fig. 6:**
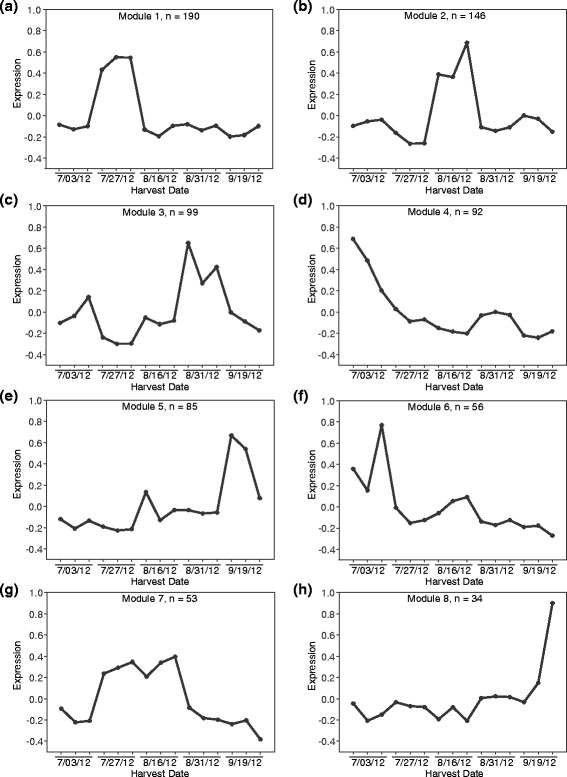
WGCNA analysis of developmental gene expression for 755 RGHs in the switchgrass cv. Summer. Shown are eight modules (**a**-**h**) that correspond to the expression patterns of 755 RGHs identified in this study over five sampling time points. The *line* represents the overall expression pattern of each co-expression module. The number of genes included in each module is represented by n

Recent studies have suggested that NB-LRR resistance genes are also differentially regulated during various developmental stages [56, 57]. In order to determine if this occurs in switchgrass, RNA-seq data from the flag leaves of field grown cultivar ‘Summer’ switchgrass plants, collected over five time points from July to September of 2012 [44], were obtained and analyzed for NB-LRR gene expression. A total of 755 of the 1011 RGHs were found to be expressed in all 3 biological replicates from at least 1 time point. These 755 RGHs were selected for Weighted Gene Correlation Network Analysis (WGCNA) which classified these genes into eight co-expression modules (Fig. 6). Modules 1, 2, and 7 (Fig. 6a, b, and g) had clear harvest/developmental stage specific expression at anthesis (7/27), seed set (8/16), and at both anthesis and seed set, respectively. Modules 4 and 6 were comprised of RGHs that were expressed at high levels at heading (7/03; Fig. 6d and f, respectively); however, module 4 showed additional expression at seed maturation (8/31) whereas module 6 showed additional expression at seed set (8/16). Co-expressed RGHs found in module 3 showed primary expression at seed maturation (8/31; Fig. 6c) and additional minor expression at heading (7/03). Finally, RGHs in modules 5 and 8 were expressed mainly at the onset of senescence (9/19; Fig. 6e and h, respectively). In general, the RGHs belonging to module 8 were expressed at a higher level than those belonging to module 5, although some variation was observed among the biological replicates.

## Discussion

The identification and validation of plant disease resistance genes is a major focus in the molecular investigations of plant-pathogen interactions. While other studies have aimed to understand the molecular mechanisms controlling switchgrass resistance to switchgrass rust [13, 30], none of these studies have mined the currently available draft switchgrass genome (v 1.1) for potential NB-LRR resistance gene homologs. In this research, a homology-based computational approach was used to identify 1011 unique RGHs in the switchgrass genome. Approximately 266 % more RGHs were identified than in a similar study that detected switchgrass RGHs from EST sequences and PCR-based cloning [30]. However, the total number of RGHs in switchgrass may change with further refinement of the switchgrass genome; although, the percentage of RGHs identified in this study is similar to rice [58].

Structural analysis of the 1011 switchgrass RGHs provided useful insights into their putative molecular functions. We identified several of the major features expected in plant NB-LRRs [20, 54] in switchgrass. Additionally, the large number of putative RGHs indicates that there is a substantial repertoire of disease resistance genes in the switchgrass genome and that a robust immunity potential is present in the event of pathogen invasion. However, further studies are needed to validate the sub-cellular localization and functions of these proteins.

The NB domain of plant R protein has been shown to act like a molecular switch and function in signal transduction pathways. In analyzing the 1011 switchgrass RGHs, 600 were found to contain a full-length NB domain while the remaining sequences were missing one or more highly conserved motifs (P-loop/WalkerA, Kinase 2, RNBS, and GLPL; [15]. This could be explained by the assembly of the switchgrass genome (v 1.1), which consists of 18 pseudomolecules and several thousand additional contigs ranging in size from 1000 to 88,021 bp [31]. Incomplete NB-LRR gene sequences in switchgrass could be a result of incomplete duplications or transversions, incomplete assembly or annotations, or may actually be pseudogenes. Some NB-LRR pseudogenes have been shown to code for non-functional or truncated protein products [59]. Interestingly, the evolution of a pseudogene at the *Pid3* gene locus has been found to promote disease in susceptible rice cultivars [60].

Sequence duplication and divergence is also prominent in NB-LRR genes. Phylogenetic analysis of the switchgrass and *Brachypodium* NB-LRRs found that the majority of the *Brachypodium* NB-LRRs have been conserved in switchgrass. Several switchgrass RGHs, including ten that were identified to contain a protein kinase domain, however, appear to have emerged after the two species diverged.

Unique domains other than the NB-LRRs identified in the switchgrass RGHs have also been identified in other species, and could play roles similar to ones reported recently in *Arabidopsis*. In *Arabidopsis* the *RRS1-R* gene encodes NB-LRR protein that contains a WRKY domain that acts as a decoy to intercept effector molecules secreted by *Pseudomonas syringae* pv. *pisi* and *Ralstonia solanacearum* [61].

Two switchgrass RGHs, Pavir.Fa01782 and Pavir.Hb00484, were found to have a C-terminal domain homologous to thioredoxin proteins. One switchgrass RGH, Pavir.Bb01048, was predicted to have a C-terminal glutaredoxin domain. Thioredoxin and glutaredoxin proteins participate in oxidation/reduction reactions and have been associated with increased disease resistance in tobacco [62, 63] and increased disease susceptibility in *Arabidopsis* [64]. Therefore, the NB-LRR containing a thioredoxin domain may function in disease resistance by reducing pathogen-induced oxidative stresses. Since glutaredoxin has been shown to promote disease resistance [64], the presence of a glutaredoxin domain in a NB-LRR disease resistance gene may function as a decoy similar to the data described by Sarris et al. [61].

A total of 5 switchgrass RGHs were predicted to contain unique domains that are involved in DNA binding. One switchgrass gene, Pavir.J03445, was found to contain a WD40 domain in the C-terminal. Plant genes that contain WD40 domains have been shown to be differentially regulated during pathogen infection, suggesting that these genes may be important regulators of defense-related responses [65, 66]. Another switchgrass gene, Pavir.J20878, which contains an N-terminal B3 DNA-binding domain, was also found to contain a C-terminal WRKY DNA-binding domain. This further supports a role for Pavir.J20878 in DNA binding and transcription regulation.

Aside from DNA-binding, several other smaller categories were identified. One switchgrass RGH, Pavir.Hb01174, is predicted to function in sugar binding. This particular RGH contains a C-terminal jacalin-like lectin binding domain. Jacalin-like lectin domains bind carbohydrates, mainly mannose and galactose, and have been shown to play an important role in disease resistance [67]. For example, the *RTM1* gene of Arabidopsis encodes a protein that contains a jacalin-like lectin domain and this protein is critical for inhibiting long-distance movement of the tobacco etch virus [68]. Additionally, three switchgrass RGHs, Pavir.Ib02384, Pavir.Ib02433, and Pavir.J18369, are predicted to function in protein trafficking and vesicle movement, a function that has not yet been demonstrated by plant disease resistance genes. These proteins contain a domain in their N-terminals that shows strong homology to a major sperm protein of nematodes. A previous report has identified a similar domain in the N-terminal region of the VAP27 protein of tomato, which has been shown to interact with the Cf9 resistance protein; however, no direct role for VAP27 in disease response has been established [69]. To our knowledge, this is the first report of a major sperm protein domain attached to the N-terminal of a NB-containing resistance gene.

Several other domains found in the switchgrass RGHs, have been linked to disease response in other plants. These include calmodulin (Pavir.Hb00190) [70, 71], WD domain (Pavir.J03445) [72, 73], and transposable elements (Pavir.Fb0150) [74, 75]. This highlights the diversity and potential for gene diversification of RGHs encoded by the switchgrass genome.

The switchgrass cultivars ‘Alamo’ and ‘Dacotah’ exhibit significantly different disease responses after exposure to switchgrass rust (*Puccinia emaculata*). ‘Alamo’ is relatively resistant to the rust pathogen whereas ‘Dacotah’ is highly susceptible [9]. Polymorphisms within RGHs may contribute to the disease resistance phenotype that is observed between ‘Alamo’ and ‘Dacotah’. In our study, we identified 2634 variants between ‘Alamo’ and ‘Dacotah’, including SNPs, MNPs, indels, and replacements. Approximately 89 % of the variants detected were SNPs. The predominance of SNPs could be attributed to the fact that single nucleotide changes in the coding regions of genes are less likely to disrupt the reading frame, which often times results in nonsense mutations. SNPs could also explain the disease response phenotypes of ‘Alamo’ and ‘Dacotah’, as they could alter the amino acid sequence of resistance genes and potentially disrupt gene function. Since these SNPs are associated with defense-related genes, they could be further developed into molecular markers for use in breeding of disease resistance.

In addition to polymorphisms within the RGHs, differential expression of NB-LRR disease resistance genes may contribute to the different phenotypes observed between the two cultivars. It is believed that *R* genes are expressed at relatively low levels in unchallenged plant cells in anticipation of pathogen attack [14]. Indeed, we found that 338 RGHs displayed expression evidence in both cultivars in an unchallenged state, supporting the idea that *R* genes are basally expressed in healthy plant cells [14]. There could be several reasons for the genes that we could not find expression evidence for. First, these genes could be expressed at extremely low levels in healthy plant cells and thus, they escaped detection at the sequencing coverage used in this study. Second, the expression of these genes could be induced upon pathogen detection and they are not basally expressed in healthy plant cells. Finally, some of these genes may be pseudogenes and may not be expressed under any conditions. Further studies are needed to evaluate the expression patterns of the switchgrass RGHs and to determine the exact role, if any, that these genes play in switchgrass disease response.

Developmental regulation of specific RGHs could also contribute to disease resistance phenotypes at different stages of plant growth. RNA-sequencing from the flag leaves of field grown cultivar ‘Summer’ provided a unique opportunity to examine switchgrass RGH expression over the course of a growing season [44]. The first group of developmentally regulated RGHs (module 1, Fig. 6a) is of particular interest since these genes are up-regulated at the end of July. The end of July and the beginning of August are optimal times for switchgrass rust infection and thus, these genes may play an important role in immediate defense responses against foliar pathogens like switchgrass rust. Correspondingly, the transcripts for these RGHs decreased over the remaining harvests, supporting the idea that these genes are involved in the earlier stages of disease response. Field-grown switchgrass plants appear to be more susceptible to switchgrass rust as they begin to flower and set seed (data not published). As displayed in modules 5 and 8, 119 of the 755 RGHs (16 % of the genes) exhibited peak gene expression in at least one biological replicate during the last sampling point (9/19). The remaining 84 % of the genes displayed peak gene expression over the first four sampled time points. Since fewer RGHs showed preferential expression during the later stages of the growing season, these results support the likelihood that switchgrass plants may utilize resources towards other processes, such as flowering and nutrient remobilization, rather than disease resistance in the later stages of development.

## Conclusion

The results of this study provide useful insight into the molecular structure, distribution, and expression patterns of members of the NB-LRR gene family in switchgrass, an important biofuel crop. Switchgrass molecular breeding is relatively recent and has been hindered by a lack of informative genomic resources. The 2347 SNPs that we identified by RNA-sequencing can potentially be developed into molecular markers, which may assist switchgrass breeders in creating new cultivars with improved disease resistance. In addition, we also identified 40 RGHs that are predicted to contain unique domains including major sperm protein domain, jacalin-like binding domain, calmodulin-like binding, and thioredoxin. NB-LRR proteins that contain unique domains may have arisen in switchgrass as a method of molecular adaptation to plant pathogens. Thus, this research can be used for homology-based methods that could identify NB-LRR disease resistance genes with these unique domains in other plant species Taken together, the results of this study provide novel findings that will aid in the identification of NB-LRR disease resistance genes and breeding for durable disease resistance in the biomass crops.

## Additional files

Additional file 1: Table S1. List of 1011 unique switchgrass proteins that are predicted to contain an NB-ARC domain (1E < -10). Table S1 lists the protein gene IDs for the 1011 putative switchgrass RGHs, the length of the predicted proteins according to Phytozome, and the amino acid sequences. (XLSX 433 kb)
Additional file 2: Table S2. List of switchgrass RGHs that were predicted to contain one or more coiled-coil (CC) domains. Table S2 contains a list of switchgrass RGHs that were predicted to contain one or more coiled-coil domains. It also includes the amino acid start location of that domain(s) and the amino acid end of that domain(s). (XLSX 23 kb)
Additional file 3: Table S3. List of 19 switchgrass RGHs that were predicted by Signal P to contain an N-terminal signal peptide and the TargetP prediction of their subcellular localization. Table S3 lists the 19 switchgrass RGHs that were predicted by Signal P to contain an N-terminal signal peptide. This list also contains the predicted subcellular location of these proteins as determined by TargetP, the amino acid start and stop location of the signal peptide, and the amino acid sequences of these proteins with the signal peptide sequence highlighted in red. (XLSX 18 kb)
Additional file 4: File S1. Manual annotation of the 1011 switchgrass RGHs. File S1 details the manual annotation of the 1011 switchgrass RGHs. The 1011 switchgrass RGHs were manually searched through for the presence of 4 conserved motifs and then separated into different categories based on if these motifs were present. The tabs at the bottom of the file correspond to the different categories and the Index tab details how these RGHs were characterized. (XLSX 439 kb)
Additional file 5: Table S4. List of 104 switchgrass RGHs that were predicted to contain a NLS using NLStradamus. Table S4 contains a list of 104 switchgrass RGH protein IDs that were predicted to contain a NLS using NLStradamus. This list also details the algorithm of NLStradamus used to predict the NLS and the score, the length of the switchgrass RGH amino acid sequence, the amino acid start and stop position of the NLS, and the putative NLS sequence. (XLSX 17 kb)
Additional file 6: Table S5. List of LRRs identified in the 1011 RGHs. Table S5 lists the 1011 switchgrass RGHs and the number of LRRs that were identified in the C-terminal of each of the protein sequences using the signature ‘LxxLxxLxx’. The C-terminal was defined as the region following the end of the NB domain. Also included in this list is the protein length of each of the RGHs, the numerical amino acid end of the NB domain, and the amino acid start position for the LRRs that were identified. (XLSX 63 kb)
Additional file 7: Figure S1. Labeled phylogenetic tree of 578 switchgrass RGHs and 116 *Brachypodium distachyon* RGHs. Figure S1 is a replica of the phylogenetic tree included in the paper but it contains the IDs of the 578 switchgrass RGHs and 116 *Brachypodium distachyon* RGHs. (PDF 820 kb)
Additional file 8: Table S6. High quality variants detected between ‘Alamo’ and ‘Dacotah’. Table S6 details the high quality variants that were detected between ‘Alamo’ and ‘Dacotah’. This table includes the chromosome and subgenome information, the region on the chromosome where the variant is located, the type and length of variant, the information for ‘Alamo’ and ‘Dacotah’ at this location, and the average base quality as determined by RNA-seq. (XLSX 166 kb)
Additional file 9: Table S7. Allele-specific PCR primers for validation of SNPs at 4 loci for ‘Alamo’ and ‘Dacotah’. Table S7 lists the allele-specific primer sequences that were used for validation of SNPs identified by RNA-seq for ‘Alamo’ and ‘Dacotah’. This table also includes the genomic location and name of gene that contains the SNP, the base position of the SNP, the expected PCR product size, and the annealing temperature used during PCR for each primer set. (XLSX 11 kb)
Additional file 10: Table S8. Conserved Primers used to validate SNPs detected between ‘Alamo’ and ‘Dacotah’. Table S8 details 12 conserved primer pairs that were used to validate SNPs detected between ‘Alamo’ and ‘Dacotah’. Also included in the table are the location of the SNP, the strand of DNA containing the SNP (+/-), and the SNP base pair for ‘Alamo’ and ‘Dacotah’. Other information that is included is the chromosome/contig start and end base pair for the primers and the expected length of the PCR product. (XLSX 11 kb)
Additional file 11: Figure S2. PCR validation of SNPs identified between ‘Alamo’ and ‘Dacotah’ using conserved primers. Figure S2 contains sequencing and alignment data from PCR validation of SNPs identified between ‘Alamo’ and ‘Dacotah’ using conserved primers. The SNP locations predicted by RNA-seq have been highlighted in yellow. (DOCX 25 kb)
Additional file 12: Table S9. List of 338 genes that were identified to be expressed in both ‘Alamo’ and ‘Dacotah’. Table S9 lists the gene IDs for the 338 genes that were identified to be expressed in both ‘Alamo’ and ‘Dacotah’. Also listed in this table is the mean reads for each cultivar, which is the average number of reads of three biological replicates mapping to the gene. A gene was considered expressed if 5 or more reads mapped to it. (XLSX 19 kb)
Additional file 13: Table S10. Cultivar specific expression of NB-LRR RGHs in switchgrass. Table S10 contains 117 genes that were found to be expressed only in ‘Alamo’ and 134 genes that were found to be expressed only in ‘Dacotah’. Means are the average number of reads mapping to the gene from three biological replicates of each cultivar. A gene was considered expressed if 5 or more reads mapped to it. (XLSX 18 kb)

## Abbreviations

bp: Base pair
CC: Coiled-coil domain
EDGE test: Empirical analysis of differential gene expression test
FDR: False discovery rate
indels: Insertions and deletions
LRR: Leucine rich repeat
ME: Module Eigengene
MNPs: Multiple nucleotide polymorphisms
NB: Nucleotide binding site
NB-ARC: Proteins containing a nucleotide binding site and domains homologous to human APAF-1, plant disease resistance proteins, and *C. elegans* CED-4 protein
NB-LRR: Nucleotide binding site- Leucine rich repeat genes/proteins
NLS: Nuclear localization signal
Q-score: Quality score
R genes: Plant disease resistance genes
RGH: Resistance gene homolog
RNA-seq: RNA-sequencing
RPKM: Reads per kilobase of transcript per million mapped reads
SMART: Simple modular architecture research tool
SNP: Single nucleotide polymorphism
TIR: Domain homologous to *Drosophila* Toll and mammalian Interleukin 1 Receptors
WGCNA: Weighted gene co-expression network analysis
