# Supplementary figures and images for: Identification, characterization, and gene expression analysis of nucleotide binding site (NB)-type resistance gene homologues in switchgrass
Source: BMC Genomics. 2016 Nov 8;17:892. doi: 10.1186/s12864-016-3201-5 (PMC5100175; doi:10.1186/s12864-016-3201-5)

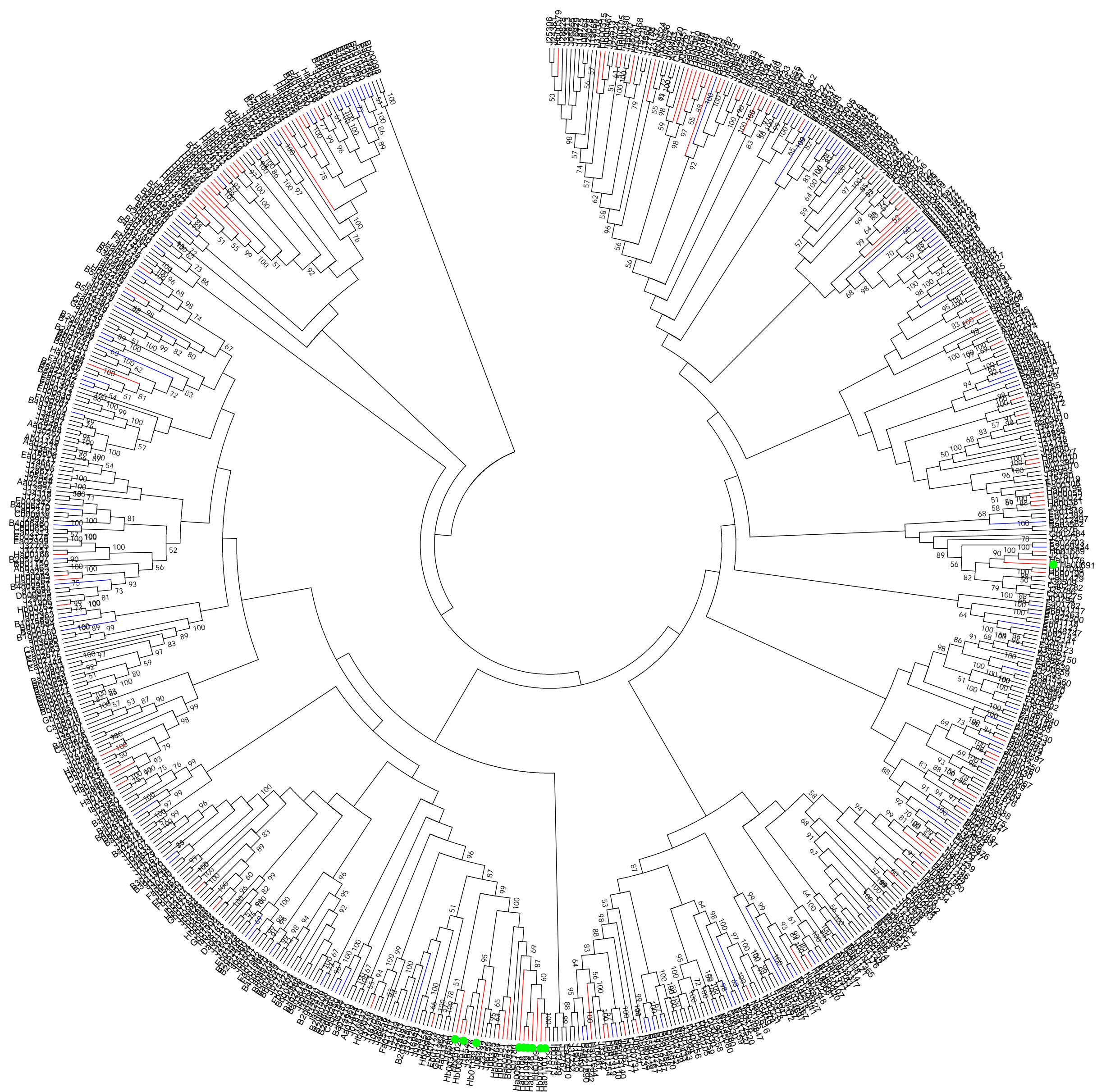

Supplement: Additional file 7: Figure S1. — Labeled phylogenetic tree of 578 switchgrass RGHs and 116 Brachypodium distachyon RGHs. Figure S1 is a replica of the phylogenetic tree included in the paper but it contains the IDs of the 578 switchgrass RGHs and 116 Brachypodium distachyon RGHs. (PDF 820 kb) [file 12864_2016_3201_MOESM7_ESM.pdf]
