# Supplementary material for: Identification, characterization, and gene expression analysis of nucleotide binding site (NB)-type resistance gene homologues in switchgrass
Source: BMC Genomics. 2016 Nov 8;17:892. doi: 10.1186/s12864-016-3201-5 (PMC5100175; doi:10.1186/s12864-016-3201-5)
Supplement: Additional file 11: Figure S2. — PCR validation of SNPs identified between ‘Alamo’ and ‘Dacotah’ using conserved primers. Figure S2 contains sequencing and alignment data from PCR validation of SNPs identified between ‘Alamo’ and ‘Dacotah’ using conserved primers. The SNP locations predicted by RNA-seq have been highlighted in yellow. (DOCX 25 kb) [file 12864_2016_3201_MOESM11_ESM.docx]

**Figure S2.** PCR validation of SNPs identified between ‘Alamo’ and ‘Dacotah’ using conserved primers.

>sh5127- Pavir.J03515- Alamo: T (shows as A in the reverse sequence)

>sh5128- Pavir.J03515- Dacotah: G (shows as C in the reverse sequence)

**Sequence is the reverse complement

sh5127 1 ---------------------TTAA---------GGCT-TATGATGAGA-TTTCTCTGCC
sh5128 1 ---------------------TTA----------GGCA--ATGATGAGA-TTTCTCTGCC

sh5127 29 A-GTTGGCTACTATAATTGAGCAACTACATTATGTCCGGGTTCTCCGGTTGCAGTTCAAT
sh5128 27 A-GTTGGCTACTATAATTGAGCAACTACATTATGTCCGGGTTCTCCGGTTGCAGTTCAAT

sh5127 88 GATGATATATTGTTGGCCAGTGTAAAAAAGTTCATCCATCTGCGGTATTTGGAACTAAGA
sh5128 86 GATGATATATTGTTGGCCAGTGTAAAAAAGTTCATCCATCTGCGGTATTTGGAACTAAGA

sh5127 148 TATACTTCTGACATGCAGAAACCTTTGCCTAAGCATATATGTGAGCTCTACCACCTACAA
sh5128 146 TATACTTCTGACATGCAGAAACCTTTGCCTAAGCATATATGCGAGCTCTACCACCTACAA

sh5127 208 ATATTAGACGTCAGACATTGGAATGGTCTAAATGATTTACCAGAGGGTATGAATAATCTT
sh5128 206 ATATTAGACGTCAGACATTGGAATGGTCTAAATGATTTACCAGAGGGTATGAATAATCTT

sh5127 268 GTGAACCTACGTTATCTGCTTGTTCCCGGGTCAGGATCATTGCACTCAAAGATATCAAAA
sh5128 266 GTGAACCTACGTTATCTGCTTGTTCCCGGGTCAGGATCATTGCACTCAAAGATATCAAAA

sh5127 328 GTTGGAGATCTTGAGTTTTTACAAGAACTGAAAGAATTCCGAGTTCAAAAAAAGGATGGC
sh5128 326 GTTGGAGATCTTGAGTTTTTACAAGAACTGAAAGAATTCCGAGTTCAAAACAAGGATGGC

sh5127 388 TTTGATATTTCACAGCTGGGGAACTTAAAAGAGATCAAAGGGTCACTTAGTATCCTTGAT
sh5128 386 TTTGATATTTCACAGCTGGGGAACTTAAAAGAGATCAAAGGGTCACTTAGTATCCTTGAT

sh5127 448 CTTGAGAATGTCACGAGCAAGGAGGAGGCGACCCGTGCAGGGATTAAACAGAAGAAACAT
sh5128 446 CTTGAGAATGTCACGAGCAAGGAGGAGGCGACCCGTGCAGGGATTAAACAGAAGAAACAT

sh5127 508 TTGAGGACACTTTCACTCTCATGGGGGAGCGCAAGCGCAAGTCCAGCTGCTATCCAGAAG
sh5128 506 TTGAGGACACTTTCACTCTCATGGGGGAGCGCAAGCGCAAGTCCAGTGCCTATCCAGAAT

sh5127 568 A
sh5128 566 A

>sh5129- Pavir.Ha00691- Alamo: A,A (presents as T and T in the reverse sequence)

>sh5158- Pavir.Ha00691- Dacotah: C,G (presents as G and C in the reverse sequence)

**Sequence is the Reverse Complement

sh5129 1 CCCGCCCCTTGTGTTAGAGTTATGTCCGAGGTCGAAATACCACAGTGTACGTAAAGACTT
sh5158 1 --CACAATTTGTGTTAGAGTTATGTTTGAGGTCG-AATACCTCAAAGTATTTAAAGATTT

sh5129 61 CATGCGGCCAATCCCATCCGGTAGCTTTGTTGCATCTTGGAGACCAAGATGCAGCAAACT
sh5158 58 CATGCGGCCAATCCCATTCGGTAGCTTTGTTGCATCTTGGAGACCAAGATGCAACAAACT

sh5129 121 CGGAAGATGGACAATATCTGATGGAACTGAAGATATTCTTGCATCTATTTCCATAGTCTC
sh5158 118 CGGAAGATGGACAATATCCGATGGAACTGAAGATATTCTTGCATCTATTTCCATAGTCTC

sh5129 181 CAAATAATGTAAACCTTGCATCTGGGCTGGTAGTTCAACCATGACATCACTTGAAATCTT
sh5158 178 TAAATAATGTAATCCTTGCATCTGGGTTGGTAGTTCAACCATGCTATCACTTGAAATCTT

sh5129 241 CAGATATTTCAGCTGAAATAATCTGCAAATTCGTGAGAGGTTCAAACTCATATGTCCATC
sh5158 238 TAAATATCTCAGCTGAAATAATCTGCAAATTCGTGAGAGGTTCAAACTCGTAGCTCCATC

sh5129 301 ATGATCGCCCCAACAATCGAGGATTAGAACTCGGAGATGCTTGAATTCCACAATTGAAGG
sh5158 298 AAGATCTCCCTGACAATCAAGGATTAGAACTCGGAGATATTTGAATTCCACAATTGAAGG

sh5129 361 CATACATTTATGGATTCCAAAGAAACCAAGCGATCGTAATTGTGGCATTGTTATTCCTGC
sh5158 358 CATACATTTATGGACTCCAAAGAAACCAAGCGATCGTAATTGTGGCATTGTTATTCCTGC

sh5129 421 TGGTTTCTTTGCATATTTGGCAGTACTGAATCGGAAGGATAGTCGATGGGCTTTCGGAGA
sh5158 418 TGGTTGCTTTGCATATTTGGCACTACTGAATCGGAAGGACAGCCGATGGGCTTTCATAGA

sh5129 481 TAGTCCTGTTATAGTTTGAGAGTAATCTATTGCAGCAAGAAATTCGTCTTCCTTGGAATT
sh5158 478 TAGTCCTGTTATAGTTTGAGAGTAATCTATTGCAGCAAGAAATTCGTCTTCCTTGGAATT

sh5129 541 ATGAGAAATAAGATCAAGTATAATATGGTGCACTGTACAGGACAATACCTCATTACTGTC
sh5158 538 ATGAGAAATAAGATCAAGTATAATATGGTGCACTGTACAGGACAACACCTCATTGCTGTA

sh5129 601 TCCAAGTTCCATAGGTTGGATCATTCCCCTGTTGACAAGCTCGTCAAAATAGCTCTCTGC
sh5158 598 TCCAAGTTCCATAGGTTGGATCATTCCCCTGTTGACAAGCTCGTCAAAATAGCTCTCTGC

sh5129 661 AATTTCTTCTGTATCTTTTCCTTCCGTTGCATTGATAAAAACCTTCTGCTATCCATTGCG
sh5158 658 AATTTCTTCTGTATCTTTTCCTTCTGTTGCATTGAT-AAAACCTTCTGCTATCATTTGCG

sh5129 721 TCA---
sh5158 717 TCAAAA

>sh5133- Pavir.J36509- Alamo: A,G,G

>sh5134- Pavir.J36509- Dacotah: C,C,T

sh5134 1 -----GACTGTTC------------------------CCTGCTGAGT-GTTACTGAGATC
sh5133 1 ----GAATTGATC--------------------------TGCTGCACTGCTACTGAGATC

sh5134 31 A-TCGCACCCTAATGCAT-GTAG-AGATGTCAAT-TGGCTAATACCATTTGGCAGGTTTG
sh5133 31 AGACGCACCCTAATGCATCGTAGCAGATGTCAATCTGGCTAATACCATTTGGCAGGTTTG

sh5134 87 TGTCACCAGGAAGACTGACGTACCGCACGCCTGATAAATGATCAATATCCAGTGGAACCT
sh5133 91 TGTCACCAGGAAGACTGAGGTACCGCAGGCCTGATAAATGAACAATATCCAGTGGAACCT

sh5134 147 CCCTTATTCTACAATCTATTATAAGTGTCTCCAAATGGTGTAAACCTTGCATCTTGGTTT
sh5133 151 CACTTATTCTAGAATCTATTATAAGTGTCTCCAAGTGTGGTAGACCTTGCATCTTGGTTT

sh5134 207 GCAGATCCAAAGTGACATTACATACTATCTCCAAATATCTC-GTCGAAACAATTGGCAGA
sh5133 211 GCAGATCCAAAGTGACATTACATACTATCTCCAAATATCTCAGTCGAAACAATTGGCAGA

sh5134 266 CAGTGGTAAGGTCGAAACTTCTGTTATTCTGGTCGCCCCAAAGATGAAGGATCATAACAT
sh5133 271 CAGTGGTAAGGTCGAAACTTCTGTTATTCTGGTCGCCCCAAAGATGAAGGATCATAACAT

sh5134 326 GAAGGAGCCGAAACTCTGCTATAGAAGGCAAAGACTTCAAGAGTCCAAAAAATGCAAGTG
sh5133 331 GAAGGAGCCGAAACTCTGCTATAGAAGGCAAAGACTTGAAGAGTCCAAAAAAGGCAAGTG

sh5134 386 ACTGAACATGCATCAGTCTTAATCTTGTGGGTTGTGCTGCATCTTCTGCATTGCCAAATT
sh5133 391 ACTGAACATGCATCAGTCTTAATCTTGTGGGTTGTGCTGCATCTTCTGCATTGCCAAATT

sh5134 446 GGAGAGACAGTCGACGAACCTTATCAGCAAGTCTTACGTTGGCCTGAGAATGATCAATAG
sh5133 451 GGAGAGACAGTCGACGAACCTTATCAGCAAGTCTTACGTTGGCCTGAGAATGATCAATAG

sh5134 506 CAGCTACGAAATTCTCTTCTATGGACTTGTACCTAATAAGATTGTGTATTATGTAGTGCA
sh5133 511 CAGCTACGAAATTCTCTTCTATGGACTTGTACCTAATAAGATTGTGTATTATGTAGTGCA

sh5134 566 CTGTACATGATAGCACCTCACCATTGCAATTGA----
sh5133 571 CTGTACATGATAGCACCTCACCTTTGCAATTGACAGA
sh5121- Pavir.Ba02315- Alamo: G

sh5122- Pavir.Ba02315- Dacotah: A

sh5122 1 ----CTGATCGCT---------------------------GAAGTGTTGGCTGGAA-GTT
sh5121 1 ----GTTTTCCTT----------------------------GAGTGTTGGCTGGAG-GTT

sh5122 29 GTTGAGAA-TGTGGTGGTAATGCACTGTGCATAAAATCCCTAAGTGGGTTATTGTGTCAT
sh5121 28 GTTGAGAA-TGTGGTGGTAATGCACTGTGCATAAAATCCCTAACTGGGTTATTGTGTCAT

sh5122 88 TCAGAAATTGGTTTATCTCAAATGGATATGCTCGTTGATGGTATCTTACCAGCACTAAGA
sh5121 87 TCAGAAATTGGCTTATCTCAAATGGATATGCTTGTTGATGGTATCTTACCAGCACTAAGA

sh5122 148 CTTTGTTATGATCTTTTGCCATCACACCTTCAGCAATGTTTCAAATTTTGTTCCTTATTC
sh5121 147 CTTTGTTATGATCTTTTGCCATCACACCTTCAGCAGTGCTTCAAGTTTTGTTCCTTATTC

sh5122 208 CCCAAAGATTATCATTTCATCAAGCATCACATTGTCCGATTATGGATAGCTCAAGGTTTT
sh5121 207 CCCAAAGATTATAATTTCATCAAGCATCACATTGTCCGATTATGGATAGCTCAAGGTTTT

sh5122 268 GTCTTCCCTGAAGAAGGTTGTCAACCAGAAGAGACTGGCCTGCATTATTTTGACGAGTTG
sh5121 267 GTCTTCCCTGAAGAAGGTTGTCAACCAGAAGAGACTGGCCTGCATTATTTCGACGAGTTG

sh5122 328 TTCTGCAGATCATTTTTTCAGCTCTCTCCCTTCCATAATGATAATGAAGATAAGTTTGTT
sh5121 327 TTCTGCAGATCATTTTTTCAGCTCTCTCCCTTCCATAATGATAATGAAGATAAGTTTGTT

sh5122 388 ATGCATGAGCTATTTCATGATTTAGCACAATCTGTTTCAAAGAATGAGTGCTTCAGATCC
sh5121 387 ATGCATGAGCTATTTCATGATTTGGCACAATCTGTTTCAAGGAATGAGTGCTTCAGATCC

sh5122 448 GAAGA—

sh5121 447 GAAGAAA

>sh5123 - Pavir.J39382- Alamo: T

>sh5124 - Pavir.J39382- Dacotah: G

sh5123 1 ---------------------------------GAAGG-AGGAGATTGGGTGCAGTTGCA
sh5124 1 ----------------AGAG---------GAGCAGTA---------TGGGTGCAGTTGCA

sh5123 27 GCAGCTGTCCAGTGTGAGTGACACTAAGTTGGGCAAGGAAGAAGATCCCTGCATCCAAGC
sh5124 27 GCAGCTGTCCAATGTGAGTGACACTAAGTTGGGCAAGGAAGAAGATCCCTGCATCCAAGC

sh5123 87 AGGGAACAAACTGCCCTCAAAACCTCTGATTGTGAGCTCTTGCAGATTGGTGTGCGGCTG
sh5124 87 AGGGAACAAACTGCCCTCAAAACCTCTGATTGTGAGCTCTTGCAGATTGGTGTGCGGCTG

sh5123 147 GAGGCTTTCAAGGACCTGCTCAACCATATTTTCTGTGTCTTCGTCTTCGTCTTCATCATC
sh5124 147 GAGGCTTTCAAGGACCTGCTCAGCCATATTTTCTGTGTCTTCGTCTTCGTCTTCATCATC

sh5123 207 AATGTTGATGCCACCATCATGCCATTGCAGTGTCAAATCCTTTAGGTGATGCTTATCCTT
sh5124 207 AATGTTGATGCCACCATCGTGCCATTGCAGTGTCAAATCCTTTAGGTGATGCTTATCCTT

sh5123 267 CAGGCCAGCCTCCTGAGCCTTGCTGCCAGTTTTGACATTTTCAAGTCCTGAAATCTGAAG
sh5124 267 CAGGCCAGCCTCCTGAGCCTTGCTGCCAGTTTTGACATTTTCAAGTCCTGAAAGCTGAAG

sh5123 327 GCAACCCCTCAGATTCTGCAAGTTTCCCAGCTCACTGACCGAGCAGTGTGACGGGTGACT
sh5124 327 GCAACCCCTCAGATTCTGCAAGTTTCCCAGCTCACTGACCGAGCAGTGTGACGGGTGACT

sh5123 387 GCTTGCATGGAAGACTGGCAGTGTCCGGAGCTCAGAAAGTGGTGCTACAA----------
sh5124 387 GCTTGCATGGAAGACTGGCAGTGTCCGGAGCTCAGAAAGTGGTGCTACAAAAGTTGCATC

sh5123 ------------------------------------------------------------
sh5124 447 AACATTGACGAGGGGGATGGGTCCAAGGGCAGGGGCACCTTGTCCCACCTATAAGTCATA

sh5123 ------------------------------------------------------------
sh5124 507 GGGCATCAGGAACGAGCCGGGCCCATGTGTTAAAGATAAGGCAAAGAAAAACAGTTTATG

sh5123 ---------------------------
sh5124 567 GAGTTATAGGACTGGAAGTGCACAAGC

>sh5125- Pavir.Cb00731- Alamo: C (presents as G)

>sh5126- Pavir.Cb00731- Dacotah: T (presents as A)

**sequence is the reverse complement

sh5125- 1 ------------------------------GCAT-CATGGGTC-AGACAGATGCA-TCTG
sh5126- 1 ------------------------------GCAT-C-TGGGTC-AGA-AGATGCA-TCTG

sh5125- 28 CGGCCTCGAAGACAAAAGGATGCCGATTGAGACAATTGCTAGTGGCAAGCCTTGACATCT
sh5126- 26 AGGCCTCGAAGACAAAAGGATGCCGATTGAGACAATTGCTAGTGGCAAGCCTTGACATCT

sh5125- 88 TTGCACAATATCTTTAGCCAGCTTCTCAAGATCTCCAGGGCACTGATTATTTTCATTATT
sh5126- 86 TTGCACAATATCTTTAGCCAGCTTCTCAAGATCTCCGGGGCACTGATTATTTTCATTATT

sh5125- 148 GGCAAAGGCCCGTTTGCAGAAGAGATCATATGCATCTTTGTTGTCCAGGGATCTGAGTTC
sh5126- 146 GGCAAAGGCCCGTTTGCAGAAGAGATCATATGCATCTTTGTTGTCCAGGGATCTGAGTTC

sh5125- 208 AAGTCGACGATCCGGTCTTGCAAGAGAAGCCACATCGCCTAATCTTGTCGTGATAACAAT
sh5126- 206 AAGTCGACGATCCGGTCTTGCAAGAGAAGCCACATCGCCTAATCTTGTCGTGATAACAAT

sh5125- 268 ACGACTTTCCCCGACATCATTGAATGAATCTTTAATATCTAAAAATGTTCGTTGCTCCCA
sh5126- 266 ACGACTTTCCCCGACATCATTGAATGAATCTTTAATATCTAAAAATGTTCGTTGCTCCCA

sh5125- 328 CAAATCATCCAATACCACAATTGTTTTGTGTTTCTTCAGTTTCTTGTTCAATATTGATCT
sh5126- 326 CAAATCATCCAATACCACAATTGTTTTGTGTTTCTTCAGTTTCTTGTTCAATATTGATCT

sh5125- 388 CAATTCTGGTGTAGCCTTGTCAGGTCCCGTTTGCTCTTCCGTGTCACCGATATTCCTAAT
sh5126- 386 CAGTTCTGGTGTAGCCTTATCAGGTCCCGTTTGCTCTTCCGTGTCACCGATATTCCTAAT

sh5125- 448 CAATGTTCTCAGTAGGGAGTCCACCGTGTACGCCTTGGACATGGCTATCCAAGCATGTGC
sh5126- 446 CAATGTTCTCAGTAGGGAGTCCACCGTGTACGCCTTGGACATGGCTATCCAAGCATGTGC

sh5125- 508 CTGGAAATCCCTCTTGGCTCTTTCATACACATTTGCAACCAGTGTAGTTTTCCCCAATCC
sh5126- 506 CTGGAAATCCCTCTTGGCTGTTTCATACACATTTGCAACCAGTGTAGTTTTCCCCAATCC

sh5125- 568 TCCCATACCAGACACGGTTATCACCGTCTTATTTTGTTCTGTGGTGTT---GTGCAGCCA
sh5126- 566 TCCCATACCAGACACGGTTATCACCATCTTATTTTGTTCTGTGGTGTT---GTGCAGCCA

sh5125- 625 ATCAGTCAGTTGATCCCTGTAGGAATTGATCCCAACAAGTTCCCCATCATTAACAAACTG
sh5126- 623 ATCAGTCAGTTGATCCCTGTAGGAATTGATCCCAACAAGTTCCCCATCATTAACAAACTG

sh5125- 685 TGGAAATCGAACTTGCGGCACAAATGCTTCAGCAGCCGCCTGCTGTGGTTTACTATAAAC
sh5126- 683 TGGAAATCGAACTTGCGGCACAAATGCTTCAGCAGCCGCCTGCTGATGGTTACTATAAAA

sh5125- 745 T-
sh5126- 743 TA

>sh5135- Pavir.J37164- Alamo: T, C

>sh5136- Pavir.J37164- Dacotah: C, G

sh5135 1 ---------------GCTGGGAG--ACACCGTCCGCC--------------CTGATGTGC
sh5136 1 --------------GGTGGGGAG--ACACCGTCCGCCG-------------CGGATGTGC

sh5135 30 TGGACGTCC--TCCGCCGCCGGCGAGGGGCTCGGGCGAGTCACGCTCGCTGCTG--CCTT
sh5136 32 TGGACATTCACTCCGCCGCCGGCGAGGGGCTCGGGCGAGTCAAGCTCGCTGCTGAGCTTG

sh5135 86 GAGGCTCTGGACCG-GCTGATGCTCGGCACGTCGAACACGCTCGCCGGCTCCTGGTCGCT
sh5136 92 GACGCTCTGGACCACGCTGATGCTCTCCGTCTCGAACGCCTATTCCGGCTCCTAGTC-CT

sh5135 145 CCGCAATGAGAGGTGGCCCGTGGAATCGAGGCTCTCCAGCGCGGCTTCGAGCGCGCCGGC
sh5136 151 TGCTGATGAGAGGTG-CCCGTTGAGTCGAGGCTCTCCAGCGCGGCCTCGAGCGCGCCGGC

sh5135 205 GTGGTTGGCGGTCAGCTCGCTGAGCTCCAGCGACCGCAGATTGGCGGCCTTGGCCAGCGG
sh5136 210 GTGGTTGGCGGTCAGCTCGCTGAGCTCCAGCGACCGGAGATTGGCAGCCTTGGCCAGCGG

sh5135 265 GTTGCCGGCGCCGCCGTCCCAGCCCCGGGGCTGCACGCCGTGCAGCGTCTGGAGGCTGGG
sh5136 270 GTTGCCGGCGCCGCCGTCCCAGCCCCGGGGCTGCACGCCGTGCAGCGTCTGGAGGCTGGG

sh5135 325 CAGGTCGCCCAGGGCGCGGCTCGGCAGCGCGAACGGCGCGACGACGTGCCGCAGCGTGGG
sh5136 330 CAGGTCGCCCAGGGCGCGGCTCGGCAGCGCGAACGGCGCGACGACGTGCCGCAGCGTGGG

sh5135 385 GATCCTCCAGAACGCCGCGGTCACCTCCAGGACGTGGTGGCGGCCGCCGAGGATGAGCGA
sh5136 390 GATCCTCCAGAACGCCGCGGTCACCTCCAGGACGTGGTGGCGGCCGCCGAGGATGAGCGA

sh5135 445 -TGCAGGTTGAA------------------------------------------------
sh5136 450 -TGAAGGTTGAAGCCCGGGACCTAGACCATGATGACGGCTTCTACCCCGCGCACAAGCCG

sh5135 ------------------------------------------------------------
sh5136 509 GGCGGATAGCTGATTGTTTTTTCAAATAGCATGCAACCTGATGTGGACACACGCCGGTAC

sh5135 ------------------------------------------------------------
sh5136 569 ACTGATCTCCCCGCAATCCCTCCTTATGGCTAGCACGACTCAAGGGGAGGGCAAAAGTAA

sh5135 -
sh5136 629 T

>sh5159- Pavir.Ib01513- Alamo: T

>sh5160- Pavir.Ib01513- Dacotah: C

sh5159 1 ---GTCTC----------------------------GCAAACC--TGTTTGAGATGATAT
sh5160 1 ---GCATC----------------------------GCAA-CC--TGTTTGAGATGATAT

sh5159 28 GGCAAGTGCTGATAACTTATTTGCAAACTTGGTAAGATATTGTTTGTGCTTTTGTCACCT
sh5160 27 GGCAAGTGCTGATAACTTATCTGCAAACTTGGTAAGATATTGTTTGTGCTTTTGTCACCT

sh5159 88 TCAAGAATCCGCATTTCGCTTAGTATGCTTTCCCACTGTTCTCTATCTCCATGAGTGTCG
sh5160 87 TCAAGAATCCGCATTTCGCTTAGTATGCTTTCCCACTGTTCTCTATCTCCATGAGTGTCG

sh5159 148 GACAAAAGCACCCCAAGAGACTTAGCAGCTAACGGGGAGCCTTGGCAGTTTGCGGCAATG
sh5160 147 GACAAAAGCACCCCAAGAGACTTAGCAGCTAACGGGGAGCCTTGGCAGTTTGCGGCAATG

sh5159 208 CTCCGCCCAATGGACTCCAACTCCAGATCATGCTGATCTCTGCTACTCCACCCTGACAAT
sh5160 207 CTCCGCCCAATGGACTCCAAATCAAGATCATCCTGGTCTCTGCTACTCCACCCTGACAAT

sh5159 268 GCATAGACTTTGAGGATTTGCCAGCATTCTTCATCCCCCAAGGGCTTTAAAGGGATGCTT
sh5160 267 GCATAGACGTTGAGGATTTGCCCTTCTTCTTCATCCCCCTTGGGCTTTAAGGGGATGCTG

sh5159 328 AGAATGGGGGAACTCATCCTAGACACTCTTTCGTGTTGAGTAGTGATGAGAACCTTGTTT
sh5160 327 AGAAAGGGGGAACTCATCCTACACACTCTTTCATGTTGAGTAATGATGATAACCTTGTTT

sh5159 388 CCTTTCTCTCCAGCTGTCAGCGAGGGCCTCAGAAATTCCCAGAACTGAAATCCATCTGCC
sh5160 387 CCTTTCTCTCCCGCTGACGGCCAGGGACTCTCAAATACCCGAAACTGATCTCCCTCTGCC

sh5159 448 CAAAGGTTATCGATCACCAACAAGAACCGCTTTTTGCGCAGGTGGCCGTGCAGCCTTTGC
sh5160 447 CAAAGGTTATCCATCACCAACCAGAACCTGTTTTTGCGCACGTGGACCCGCTGCCGTTGC

sh5159 508 TGCAATATGTCCAAGCTTAGAAGCTCACATTTTTTACTGGTCACAGCTTCGATGATCATC
sh5160 507 TGCAATATGTTCAAACTTACAAGCTCATATTTTTTACTGGCTACAATTTCCATGATGATC

sh5159 568 TTGGTGGCAGTCCTCACATCACACGTATCTGGTAACCAAACCCAAATCCTGTCAGTGAAG
sh5160 567 TTGGTGGCCACCCTCACATCATCTGGATACGGTAACCAAACCCAGATCCTGACAAAGAAT

sh5159 628 AAGTTTGCTACCTTAGGATCGTTGTGGACTAACCGTGCCAGTGCTGTCTTCCCAATGCCT
sh5160 627 AATTTTGCTACCTTAGGATCGATGTGGACTAACCGTGCCGTCGTTGTCTTCCCAGTGCCT

sh5159 688 GCCGTTCCCCAAATGGAAACCACAGGGAGGTCCATGCCTGCATTGTCAGAAACCAGAGCA
sh5160 687 GCCGTTCCCCAAATGGAAACCACGTGGACGTCCGTGCCTGCATAGACAGAAACCAGAACA

sh5159 748 CGGACGATCTGCTCCTTCTCCTCGTCGCGGCCAATGGGCCTCTCGTCGCGATGCGCGGCA
sh5160 747 ATGACGATCTGCTCCTTCTCGTCGTCGCGGCGAATGTACCTCTCATCGGCATGCGCGTCA

sh5159 808 GCTTCAGGGGAGCCATGAATA
sh5160 807 GCGACAAAA-AGCCAT-----
